# Glucocorticoid-induced microRNA-511 protects against TNF by down-regulating TNFR1

**DOI:** 10.15252/emmm.201405010

**Published:** 2015-05-20

**Authors:** Leen Puimège, Filip Van Hauwermeiren, Sophie Steeland, Sara Van Ryckeghem, Jolien Vandewalle, Sofie Lodens, Lien Dejager, Sofie Vandevyver, Jan Staelens, Steven Timmermans, Roosmarijn E Vandenbroucke, Claude Libert

**Affiliations:** 1 VIB Inflammation Research CenterGhent, Belgium; 2 Department of Biomedical Molecular Biology, Ghent UniversityGhent, Belgium

**Keywords:** glucocorticoids, miRs, receptor regulation, sepsis, TNF

## Abstract

TNF is a central actor during inflammation and a well-recognized drug target for inflammatory diseases. We found that the mouse strain SPRET/Ei, known for extreme and dominant resistance against TNF-induced shock, displays weak expression of TNF receptor 1 protein (TNFR1) but normal mRNA expression, a trait genetically linked to the major TNFR1 coding gene *Tnfrsf1a* and to a locus harbouring the predicted TNFR1-regulating miR-511. This miRNA is a genuine TNFR1 regulator in cells. In mice, overexpression of miR-511 down-regulates TNFR1 and protects against TNF, while anti-miR-511 up-regulates TNFR1 and sensitizes for TNF, breaking the resistance of SPRET/Ei. We found that miR-511 inhibits endotoxemia and experimental hepatitis and that this miR is strongly induced by glucocorticoids and is a true TNFR1 modulator and thus an anti-inflammatory miR. Since minimal reductions of TNFR1 have considerable effects on TNF sensitivity, we believe that at least part of the anti-inflammatory effects of glucocorti-coids are mediated by induction of this miR, resulting in reduced TNFR1 expression.

See also: **J-M Cavaillon *et al*** (August 2015)

## Introduction

Acute systemic inflammation is a hallmark of many severe conditions, such as sepsis, severe burns, haemorrhage, ischaemia/reperfusion and others. Although this inflammation serves to remove invading micro-organisms and to restore homeostasis, an unbalanced regulation of the response causes systemic inflammatory response syndrome (SIRS), which is often fatal. SIRS is coordinated by a few powerful pro-inflammatory cytokines, including tumour necrosis factor (TNF). TNF activates the expression of many genes involved in cytokine production, leucocyte infiltration, blood pressure reduction and coagulation. It also induces cell death and wound healing and is involved in antibacterial immunity (Puimege *et al*, 2014; Van Hauwermeiren *et al*, 2011). TNF injection in mammals is a validated model system because TNF injection of microgram doses causes a lethal systemic inflammation, which is mediated by several secondary cytokines, such as interleukin-1 (IL-1), IL-17 and interferons, so inhibition of these cytokines leads to some protection against TNF (Huys *et al*, 2009; Takahashi *et al*, 2008). TNF binds two receptors, TNFR1 and TNFR2, the former of which is constitutively expressed on most cells, while the latter is inducible and has a more restricted expression pattern. The major activities of TNF (induction of inflammation and cell death) are mediated by TNFR1, while TNF’s immune-regulatory effects, for example on regulatory T cells, DC subtypes and myeloid-derived suppressor cells, are mediated by TNFR2 (Okubo *et al*, 2013; Zhao *et al*, 2012). Anti-TNF therapy, which is used for inflammatory bowel diseases, rheumatoid arthritis and psoriasis, limits both TNFR1 and TNFR2 signals. Therefore, part of the side effects of anti-TNF therapies (e.g. appearance of psoriatic-like lesions) might be prevented by selective inhibition of TNFR1 (Van Hauwermeiren *et al*, 2011). We recently demonstrated that TNFR1 is an interesting drug target by showing that reducing TNFR1 expression by 50% (TNFR1^+/−^ mice) led to total protection against TNF-induced SIRS and TNF-induced intestinal barrier dysfunction (Van Hauwermeiren *et al*, 2013, 2014).

The search for new therapeutic targets for SIRS and other TNF-mediated diseases should include investigation of the poorly understood regulation of the TNFR1 coding gene (*Tnfrsf1a*), located on distal chromosome 6 in the mouse genome (Puimege *et al*, 2014). The absence of canonical TATA and CCAAT boxes and the high GC content have been associated with the promoters of housekeeping genes (Kemper & Wallach, 1993). However, functional binding sites for C/EBP (Bristol *et al*, 2009) and NF-κB (Baxter *et al*, 2006) are present. The regulation of TNFR1 is best known at the post-transcriptional level, namely the shedding of TNFR1 by TACE/ADAM17 (Black *et al*, 1997). TNFR1 shedding and the resultant acute decrease in the number of receptor molecules on the cell surface might transiently desensitize cells to TNF action (Xanthoulea *et al*, 2004). In addition, the pool of soluble TNFR1 generated by shedding could function as a physiological attenuator of TNF activity by competing for the ligand with the cell surface receptors (Aderka, 1996). In humans, mutations affecting TNFR1 shedding have been linked with the development of TRAPS (TNF receptor-associated periodic syndromes) (Huggins *et al*, 2004). Additionally, mice expressing a non-sheddable TNFR1 are sensitive to TNF-induced and TNF-mediated inflammation (Xanthoulea *et al*, 2004).

Glucocorticoids (GCs) are well-known anti-inflammatory molecules that are strongly up-regulated by the hypothalamus-pituitary-adrenaline axis (HPA) during inflammation as a negative-feedback loop (Van Bogaert *et al*, 2011). GCs function by binding to the ubiquitously expressed GC receptor, GR. Synthetic GCs such as dexamethasone (DEX) are effective against many inflammatory diseases and against TNF-induced SIRS (Vandevyver *et al*, 2012b). The anti-inflammatory mechanism of action of GCs has long been thought to be based on direct inhibition of pro-inflammatory transcription factors such as NF-κB. But recent work strongly indicates that formation of GC-stimulated GR dimers followed by gene induction is essential, thereby suggesting that GC/GR-induced genes (GRE genes) perform anti-inflammatory actions (Vandevyver *et al*, 2012a). Several GRE genes with anti-inflammatory functions have been described, such as those coding for GILZ and MKP-1 (Pinheiro *et al*, 2013).

We previously demonstrated that the mouse strain SPRET/Ei (S) displays an extreme and dominant resistance against TNF-induced SIRS and that this trait is linked to a locus on proximal chromosome 2 and one on distal chromosome 6, the latter containing *Tnfrsf1a* (Staelens *et al*, 2002). Here, we report that despite the sequence variations in the S *Tnfrsf1a* gene, this TNFR1 is fully functional but its expression is substantially weaker than in control C57BL6 (B) mice. This difference is observed at the protein level, but not the mRNA level. This trait is also linked to proximal chromosome 2 and distal chromosome 6, suggesting a strong correlation between low TNFR1 expression level and TNF resistance. The locus on chromosome 2 contains miR-511, which we here establish as a genuine TNFR1 regulator, and which is significantly up-regulated in S mice. We show that delivery of miR-511 to mice down-regulates TNFR1 protein and protects against TNF, as well as against endotoxic shock and lethal hepatitis, and that anti-miR-511 up-regulates TNFR1 and sensitizes for TNF, both in B and in S mice, breaking the resistance of S mice to TNF. We also found that the TNF resistance of S mice is completely dependent on the overactive HPA of these mice and that the expression of miR-511 and, hence, TNFR1 is regulated by GCs. We hypothesize that GCs protect against TNF-induced lethality partly by induction of miR-511 and consequent down-regulation of TNFR1.

## Results

### SPRET/Ei mice have a low expression of a functional TNFR1

**Figure 1:**
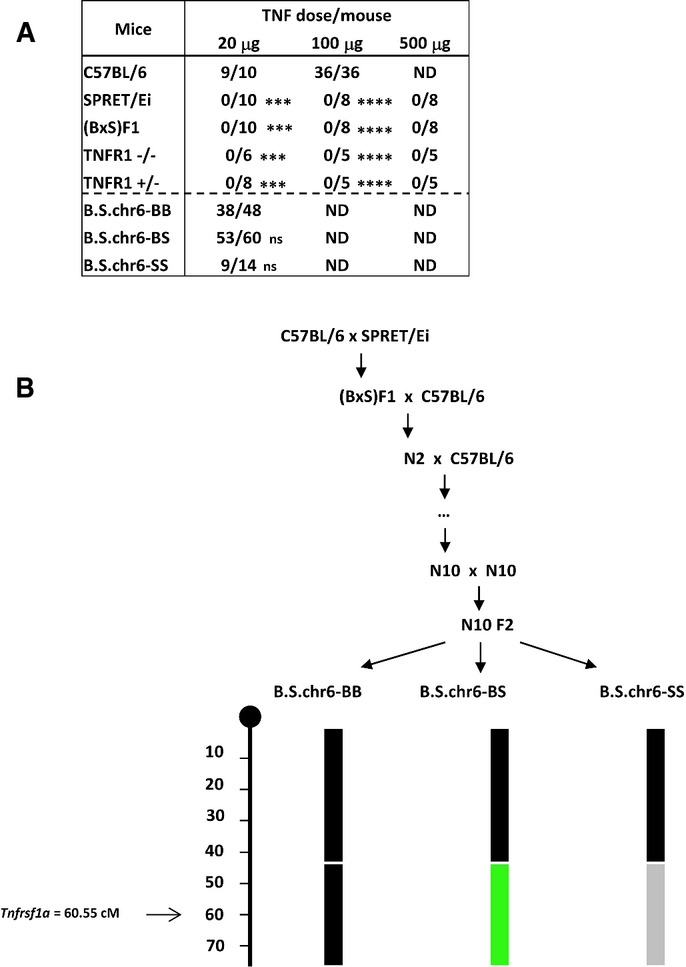
Lethal response of different genotypes to increasing doses of TNF and generation of consomic mice Mice were injected with three different doses of TNF and mortality was observed for 96 h. Ratios of deaths/total are displayed. Statistical significance of the differences in survival was calculated relative to the B group or the B.S.chr6 BB group. Student’s *t*-test using unpaired comparisons and two-tailed analysis. ***P < 0.001 and ****P < 0.0001.

We previously showed that SPRET/Ei (S) mice, unlike C57BL/6 (B) mice, are extremely resistant to lethal inflammatory shock induced by TNF (Staelens *et al*, 2002). We confirm that the LD_100_ of a single injection of TNF is > 500 μg in S mice and ∼20 μg in B mice. Mice resulting from a cross of B and S, (BXS)F1 mice, are also resistant (Fig1A). This resistance is linked to proximal chromosome 2 (0–40 cM with a peak at 15 cM) and distal chromosome 6 (40–70 cM with a peak at 46 cM). With this mapping resolution, the gene encoding TNFR1, *Tnfrsf1a* (60.55 cM), is a probable candidate resistance gene on the chromosome 6 locus (Staelens *et al*, 2002). Sequence analysis showed that the TNFR1 proteins of S and B mice differ by 10 AA (Supplementary Fig S1). By backcrossing the S genome into a B background for 10 generations and selecting for the distal part of chr6 using polymorphic markers located in and around the *Tnfrsf1a* locus, and then intercrossing, we generated 99.9% B consomic mice, harbouring 40 cM of distal chr6 with either two copies of B *Tnfrsf1a* (B.S-chr6-BB), two copies of S *Tnfrsf1a* (B.S-chr6-SS) or one copy of each (B.S-chr6-BS) (Fig1B). None of these mice showed any TNF resistance when injected with 20 μg TNF, suggesting that the S TNFR1 protein is equally functional as the B TNFR1 to induce lethal inflammation, when brought into a B background (Fig1A).

Breeding scheme for the generation of B.S.chr6 consomic mice for the distal part of chr6. After N10 generations of backcrossing, an intercross was performed resulting in progenies carrying at least 40-cM-sized haplotypes of B homozygosity (black), BS heterozygosity (green) or S homozygosity (grey). The location of the *Tnfrsf1a* gene is indicated.

### Low protein expression and normal mRNA of TNFR1 in S mice

**Figure 2:**
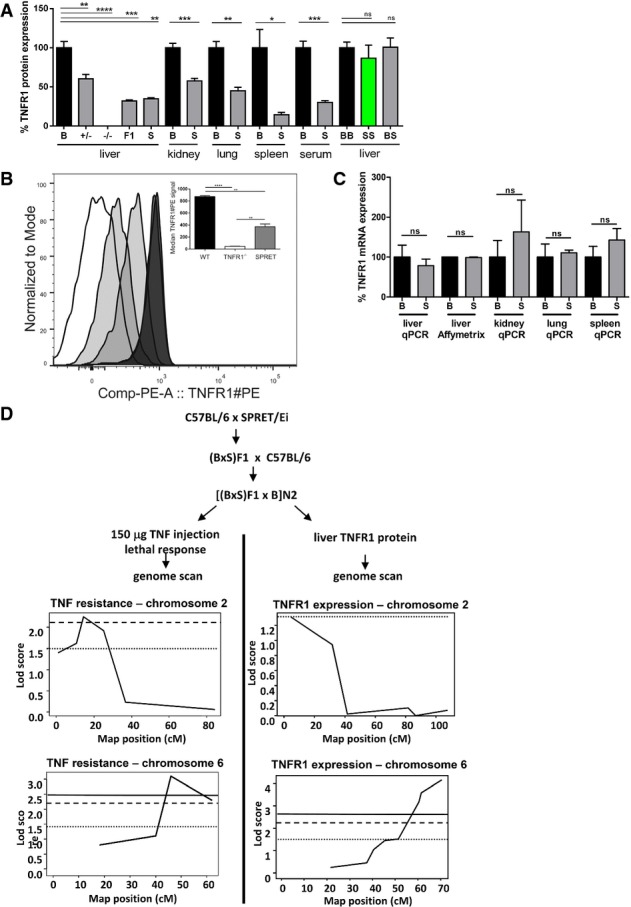
Reduced TNFR1 protein levels in S mice and genetic linkage study of this trait TNFR1 protein levels in serum and tissues of different mouse groups (*n *= 4–13). TNFR1 protein levels were measured by ELISA and compared to B, the levels of which were set as 100% for each tissue. From left to right: B mice (black), TNFR1^+/-^ mice (grey), (BxS)F1 mice (grey), S mice (grey); B.S-chr6-BB (black), B.S-chr6-SS (green) and B.S-chr6-BS (grey).

To further investigate the genetic link between TNF resistance and the distal chr6 locus, and because we had found that TNFR1^+/−^ mice, expressing 50% TNFR1, are completely resistant to TNF over a very large dose range (Van Hauwermeiren *et al*, 2013, 2014), we measured TNFR1 protein in several tissues of B and S mice and found that S mice express significantly less TNFR1 protein in all tested organs (Fig2A). This trait was dominant, and the biggest difference from B levels was in the spleen. We also found reduced soluble TNFR1 in the serum of S mice, ruling out increased shedding of soluble TNFR1 in S mice. We confirmed the low TNFR1 protein expression by measuring membrane-bound TNFR1 by FACS on splenic neutrophils. Again, TNFR1 levels on S cells were significantly lower than on B cells (Fig2B). Furthermore, there was no difference in TNFR1 protein level in the three types of consomic mice, suggesting that the TNFR1 ELISA detected B and S TNFR1 equally well, that the lack of TNF protection of the consomic mice was reflected in normal TNFR1 levels, and that the regulation of TNFR1 protein levels is not regulated by distal chr6 in *cis*, but are regulated in *trans*. However, measurement of TNFR1 mRNA levels in B and S tissues by qPCR using conserved primers revealed no differences (Fig2C). To further confirm the relation between TNF resistance and TNFR1 protein levels in S mice, we performed a new BSB genetic backcross, generating 214 N2 mice, in which we measured liver TNFR1 protein and performed genotyping using polymorphic markers. Clear linkage with broad peaks was found between low TNFR1 protein levels and proximal chr2 (0–40 cM) and distal chr6 (40–70 cM) (Fig2D). These are exactly the same sub-chromosomal regions that were linked with TNF resistance (*P* = 0.01 that both traits are linked to the same chromosomes). The location of the gene encoding TNFR1 (*Tnfrsf1a*) on distal chromosome 6 (60.55 cM) suggests that both traits are associated mainly with TNFR1 regulation. When studying epistatic interaction between the chr6 and chr2 loci in terms of TNFR1 protein regulation trait, we found (i) that the N2 mice with low TNFR1 protein level in liver (< 300 ng/mg protein) that were BS heterozygous for distal chr6 (D6Mit113), significantly more mice were BS on proximal chr2 (D2Mit359) (*n* = 50) than BB (*n* = 26) (*P* = 0.0487, chi^2^) and (ii) that the N2 mice with high TNFR1 protein level in liver (> 300 ng/mg protein) that were BB heterozygous for distal chr6 (D6Mit113), significantly more mice were BB on proximal chr2 (*n* = 36) than BS (*n* = 14) (*P* = 0.0241, chi^2^).

TNFR1 expression in splenic neutrophils of wild-type, TNFR1 KO and SPRET mice determined by FACS. Neutrophils were defined by flow cytometry (FACS) as SSC^high^CD11b^+^Ly6G^high^Ly6C^mid^ and TNFR1^+^ cells from different strains were plotted in a histogram. In the insert, the median of TNFR1-PE signals were plotted and this shows that SPRET mice have an intermediate TNFR1 expression compared to WT mice.

TNFR1 mRNA levels measured by qPCR in organs of B (*n *= 3) and S (*n *= 4). Affymetrix microarray data from hepatocytes (Affy Mo Gene 1.0 ST Array).

Backcross experiments in mice to determine genetic linkage between TNF resistance (left) and TNFR1 liver protein levels (right). QTL mapping of lethal response (*n *= 178) was based on the data previously obtained and discussed by Staelens *et al* (2002), but the data were re-analysed in the current study using R/qtl software. TNFR1 protein level in the liver of N2 mice (*n *= 214). For both TNF resistance and TNFR1 levels, the LOD scores show a QTL on chr2 (TNF resistance 15 cM, LOD* *= 2.25 and TNFR1 levels 5 cM, LOD* *= 1.31) and chr6 (TNF resistance 46 cM, LOD* *= 3.11 and TNFR1 levels 70 cM, LOD* *= 4.15). Horizontal lines in the figures represent LOD values that are suggestive (dots), significant (stripes) and highly significant (full line). Data information: Data are presented as mean ± SE. Student’s *t*-test using unpaired comparisons and two-tailed analysis. *P < 0.05, **P < 0.01, ***P < 0.001, ****P < 0.0001.

### MiRNA-511 is a predicted TNFR1 regulator and is stronger expressed in SPRET/Ei

**Figure 3:**
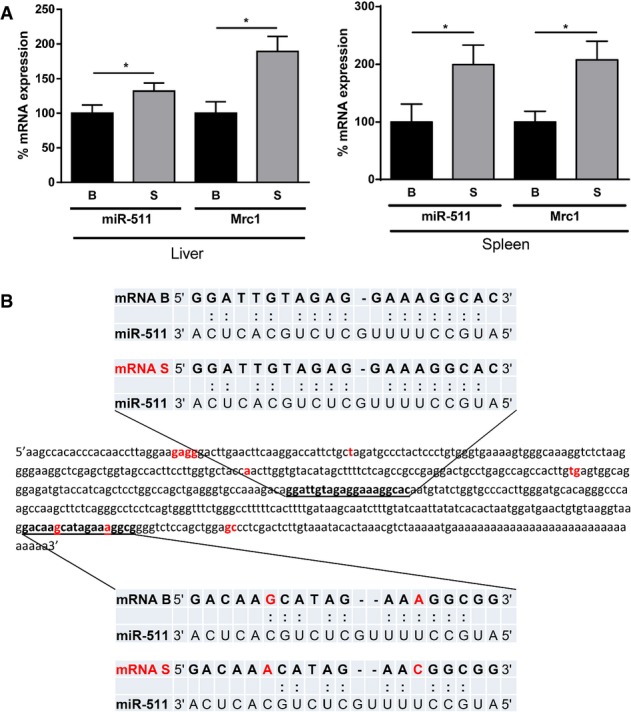
miR-511 levels, sequence and target sequences in B and S mice miR-511 and *Mrc1* mRNA levels in liver (left panel) and spleen (right panel) in B (*n *= 5) and S (*n *= 5) mice. mRNA levels were measured by qPCR and levels detected in B mice were set as 100%. Significance of the differences was studied by Student’s *t*-test, *P < 0.05. Data are presented as mean ± SE.

The regulation of TNFR1 protein but not mRNA in *trans* could imply miRNA-based regulation, and based on the linkage data, we hypothesized that a miR on proximal chr2 might be involved. Using MiR Walk (http://www.umm.uni-heidelberg.de/apps/zmf/mirwalk/) and the transcript *Tnfrsf1a*-001 (ENSMUST00000032491), we ran 10 different prediction programs and found a group of 18 miRs that possibly target the *Tnfrsf1a* gene (Supplementary Fig S2). We found two miRs on chr2, namely miR-296 (predicted only twice and located distally on 97.9 cM) and miR-511 (predicted by 6 algorithms and located at 10.5 cM). miR-511, located in intron 5 of the *Mrc1* gene, has been suggested to be strictly co-expressed with *Mrc1,* encoding the C type 1 mannose receptor (Squadrito *et al*, 2012). By qPCR, we measured miR-511 and *Mrc1* expression in liver (an essential TNF target organ) and spleen (because the TNFR1 level was very low in this organ) of naive B and S mice and found significantly stronger expression of both RNAs in both organs of S mice (Fig3A). Analysis of the sequences of the mature miR-511 in B and S revealed no differences. But comparison of the 3′ UTR of the *Tnfrsf1a* gene revealed two possible miR-511 target sequences, one of which is identical between S and B and the other one showing two nucleotide differences that interfere with the complementarity with the miR-511 seed sequence (Fig3B).

3′ UTR of the *Tnfrsf1a* gene of B mice. The single nucleotide polymorphisms (SNPs) found in the sequence of S mice are shown in red. miR-511 was predicted by the program MicroSNiPer (http://cbdb.nimh.nih.gov/microsniper/) to have two target sequences, shown in bold and underlined. The most 3′ target sequence contains two nucleotide differences (in red) in S (lower blue panels) compared to B (upper blue panels), leading to different base pairing with miR-511, as shown in the lower panel.

### MiRNA-511 is a genuine TNFR1 regulator and has therapeutic potential in mouse models

**Figure 4:**
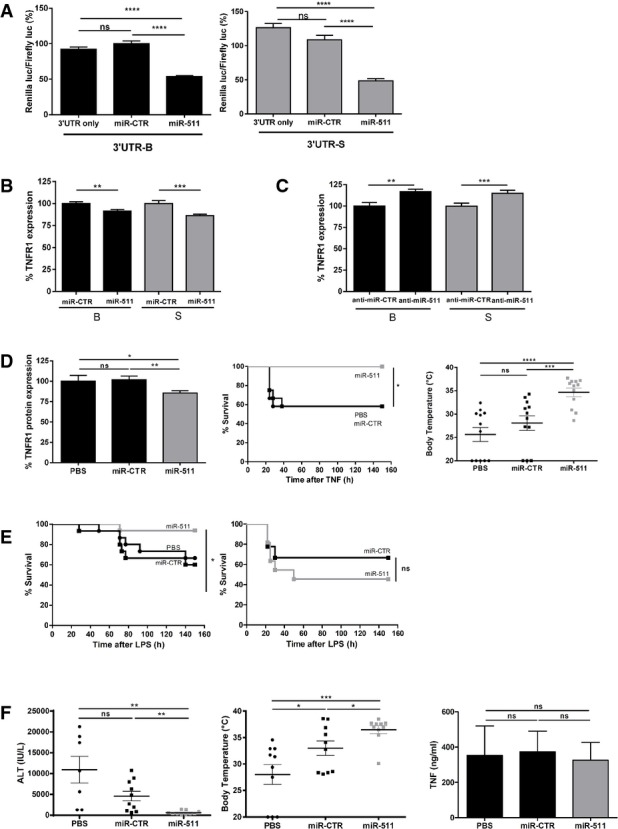
miR-511 is a genuine regulator of TNFR1 and has therapeutic potential Inhibition of *Rluc* activity of the psiCHECK-*Tnfrsf1a*-3′UTR reporter plasmid with B sequence (black, left panel) and S sequence (grey, right panel) by miR-511 transfection in HEK-293T cells (*n *= 7–8).

To study whether miR-511 is a genuine *Tnfrsf1a* regulator at the 3′ UTR level in both B and S, we generated a reporter plasmid in which the *Renilla* luciferase gene is controlled by the TNFR1 3′ UTR. Co-transfection of HEK293T cells with pre-miR-511 and the reporter plasmid significantly reduced luciferase expression after 48 h in both the B and S reporters (Fig4A). Similarly, when B and S MEF cultures were transfected with miR-511 and control miRs, cellular TNFR1 protein levels were significantly reduced 24 h later in both B and S MEFs (Fig4B). Similar results were obtained using primary hepatocytes of B and S (not shown). Furthermore, LNA-locked anti-miRs specifically inhibiting miR-511 significantly up-regulated TNFR1 in both B and S MEFs 24 h after transfection (Fig4C). We then evaluated whether miR-511 can regulate TNFR1 and the response to TNF *in vivo*. We injected pre-miR-511 under control of the CMV promoter in mice using hydrodynamic tail vain injection, which delivers plasmids to the liver (Liu *et al*, 1999). After 24 h, hepatic TNFR1 protein was measured, mice were injected with 20 μg TNF, and hypothermia and lethal shock were studied. miR-511 injection led to a 20% reduction in TNFR1 protein expression, and this reduction was associated with significant resistance to TNF-induced hypothermia and mortality (Fig4D). To study the specificity and therapeutic potential of miR-511 in mice, we investigated whether miR-511 could protect mice against LPS-induced endotoxemia, a model in which TNF is centrally involved (Tracey *et al*, 1988; Vandenbroucke *et al*, 2013). After hydrodynamic tail vein injection of the miR-511-encoding plasmid, B mice were indeed significantly protected against an LD_50_ of LPS, that is 200 μg/mouse (Fig4E). Because miR-511 was described as a regulator, under certain conditions, of TLR4, the LPS receptor (Tserel *et al*, 2011), we studied whether the protection provided by miR-511 against LPS is mediated by TNFR1 regulation. We have shown before that TNFR1 plays a role in LPS-induced lethal endotoxemia using TNFR1 KO mice (Vandenbroucke *et al*, 2013). Here, we studied the protective effect of miR-511 against an LD_50_ of LPS in TNFR1 KO mice, that is 500 μg per mouse. As shown in Fig4E, miR-511 did no longer protect against LPS in the absence of TNFR1. Since TNF is also an essential mediator in several forms of acute hepatitis (Hasselblatt *et al*, 2007), we evaluated the therapeutic potential of miR-511 in the concanavalin (ConA) model of acute, TNF-mediated hepatitis (Hasselblatt *et al*, 2007). Twenty-four hours after hydrodynamic pretreatment of mice with PBS, control miR or miR-511, mice were injected with ConA, leading to TNF release, cell death (ALT release) and hypothermia. Despite some minor effects of the control miRs, miR-511 provided significant protection against ALT release and hypothermia, without affecting the amount of TNF produced (Fig4F). These results suggest that TNFR1 reduction by miR-511 has protective effects in mouse models in which TNFR1 is implicated.

TNFR1 protein levels in B MEF cultures (black, *n *= 18) and S MEF cultures (grey, *n *= 24) 24 h after transfection with miR-511 or miR-CTR. TNFR1 protein levels were measured in cell lysates by ELISA.

TNFR1 protein levels in B MEF cultures (black, *n *= 18) and S MEF cultures (grey, *n *= 18) 24 h after transfection with anti-miR-511 or anti-miR-CTR. TNFR1 protein levels were measured in cell lysates by ELISA.

*In vivo* effect 24 h after hydrodynamic injection of B mice with plasmids expressing miR-511 (grey, *n *= 19), miR-CTR (black, *n *= 19) or PBS (black, *n *= 8). Liver TNFR1 protein levels (left panel) were measured by ELISA. Twenty-four hours after plasmid injection, mice were injected with 25 μg TNF. All mice pretreated with miR-511 (grey, *n *= 12) survived, while 50% of the mice pretreated with miR-CTR (■, black, *n *= 12) or PBS (●, black, *n *= 12) died from TNF injection (middle panel). Twenty-four hours after injection of TNF, body temperatures of mice pretreated with miR-511 were significantly higher than those of the miR-CTR and PBS groups (right panel) (all groups *n *= 10).

Survival of B mice and TNFR1^−/−^ mice injected with LPS, 24 h after miR-511 or miR-CTR hydrodynamic plasmid injection. B mice pretreated with miR-511 (grey, *n *= 16) were significantly protected against 200 μg LPS compared to miR-CTR-pretreated mice (■, *n *= 15) or PBS-pretreated mice (●, *n *= 15) (left panel). No difference in survival was found between TNFR1^−/−^ mice pretreated with miR-511 (grey, *n *= 11) or with miR-CTR (■, *n *= 9) against 500 μg LPS (right panel).

Effect of hydrodynamic injection of plasmids expressing miR-511, miR-CTR or PBS on ConA-induced hepatitis in B mice (all groups *n *= 10). A total of 360 μg ConA was injected i.v. 24 h after plasmids and 8 h later mice were analysed: liver damage (serum ALT levels, left panel); body temperatures of mice 8 h after ConA injection (middle panel); TNF levels (right panel) 2 h after injection of ConA revealed similar concentrations of TNF in the three groups. miR-511-injected mice were significantly protected in the ConA model. Data information: Data are presented as mean ± SE. Survival curves (Kaplan–Meyer plots) were compared by a log-rank test, and final outcomes were compared by a chi-square test. Differences between groups were assessed by Student’s *t*-test using unpaired comparisons and two-tailed analysis. *P < 0.05, **P < 0.01, ***P < 0.001, ****P < 0.0001.

**Figure 5:**
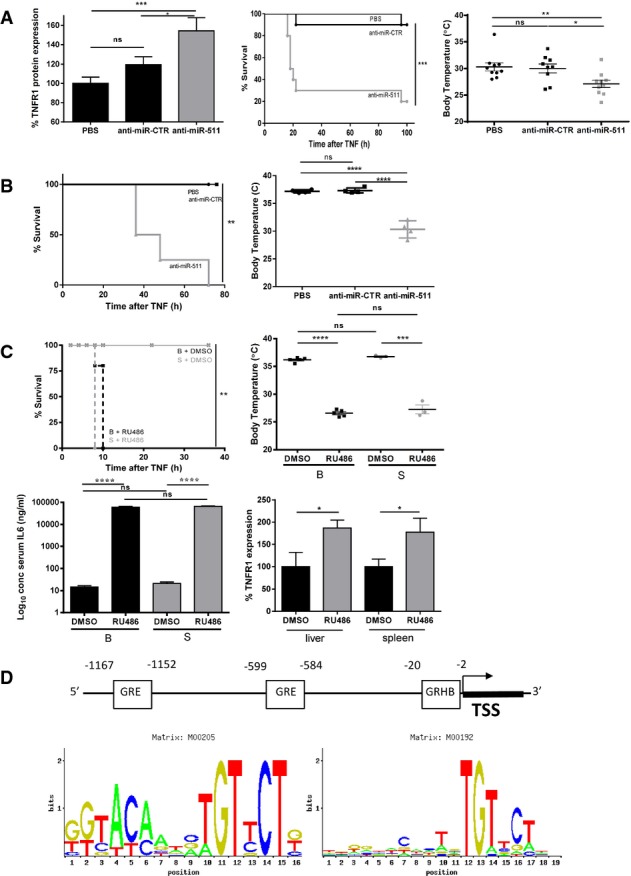
Inhibition of miR-511 and GR sensitizes for TNF-induced SIRS *In vivo* effect 24 h after hydrodynamic injection of B mice with plasmids expressing anti-miR-511 (*n *= 9), anti-miR-CTR (*n *= 9) or PBS (*n *= 10). TNFR1 protein levels in the liver were measured by ELISA 24 h after hydrodynamic injection (left panel). Survival of B mice injected with 20 μg TNF, 24 h after hydrodynamic injection (middle panel). Mice pretreated with anti-miR-511 (grey, *n *= 10) were significantly sensitized for TNF-induced SIRS compared to control groups and had the largest drop in body temperature (right panel) 12 h after injection of 25 μg TNF.

To explore further whether miR-511 is linked with TNFR1 expression and the response to TNF, we delivered LNA-locked anti-miRs by hydrodynamic injection to B mouse livers and studied TNFR1 levels and TNF response 24 h later. miR-511-specific anti-miR led to about 50% increase in expression of TNFR1 in livers (Fig5A), and to a significant sensitization of B mice to TNF as measured by lethality and hypothermia (Fig5A). Similar experiments performed on S mice resulted in significant sensitization towards an otherwise non-lethal dose of TNF in these mice, at the level of both survival and hypothermia (Fig5B). These data suggest that inhibiting miR-511 leads to loss of the robust TNF resistance of S mice. Similar data were obtained in TNF-resistant (BxS)F1 mice (Supplementary Fig S3).

*In vivo* effect 24 h after injection of S mice with plasmids expressing anti-miR-511 (▲, *n *= 4), anti-miR-CTR (■, *n *= 4) or PBS (●, *n *= 4). Survival of S mice injected with 400 μg TNF. Mice pretreated with anti-miR-511 were significantly sensitized for TNF compared to both control groups and showed the largest drop in body temperature (right panel) of all groups.

Inhibition of TNF resistance of S mice by RU486. B and S mice (all groups *n *= 5) were injected with 5 μg TNF (= LD_100_ in RU486-pretreated mice from preliminary experiments), 30 min after injection of RU486 or DMSO control. Both B (black dashed line) and S mice (grey dashed line) pretreated with RU were significantly sensitized for TNF lethality compared to DMSO/TNF controls (full lines) (top left panel) and for TNF-induced hypothermia measured 6 h after injection of 5 μg TNF. IL-6 levels 6 h after injection of TNF were equally high in RU486-sensitized B and S mice. RU486 injection in S mice (*n *= 4) led to significantly increased TNFR1 protein expression in liver and spleen 6 h later compared to DMSO vehicle-treated S mice (*n *= 4).

Two GRE elements (ConTra matrix M00205) were identified in the 5′ region of the *Mrc1* gene at −1,167 to −1,152 and −599 to −584 relative to the TSS. One GR half binding site (ConTra matrix M00192) was found at −20 to −2 relative to the TSS. Data information: Data are presented as mean ± SE. Survival curves (Kaplan–Meyer plots) were compared by a log-rank test, and final outcomes were compared by a chi-square test. Differences between groups were assessed by Student’s *t*-test using unpaired comparisons and two-tailed analysis. *P < 0.05, **P < 0.01, ***P < 0.001, ****P < 0.0001.

### SPRET TNF resistance is dependent on miR-511 and on the HPA

We then analysed the regulation of miR-511 in mice. We have previously shown that S mice have an overactive HPA, producing high basal levels of corticosterone (CS) and hence high basal levels of numerous GR-inducible (GRE-element containing) genes (Dejager *et al*, 2010). We here first validated the high basal HPA activity in S compared to B mice (all groups *n* = 6) and measured CS in serum, 2 h after 10 μg TNF or 200 μl PBS. The obtained levels were 89.33 ± 28.46 ng/ml (B, PBS), 377.83 ± 153.15 ng/ml (B, TNF), 192.66 ± 94.70 ng/ml (S, PBS) and 266.50 ± 84.83 ng/ml (S, TNF).

We investigated whether the TNF resistance of S mice depends on the HPA by pretreating S mice with RU486, an irreversible inhibitor of GR. The strong TNF resistance of S mice was completely abolished when GR was blocked by RU486, and S and B mice became equally sensitive, as measured by death from a low dose of 5 μg TNF, hypothermia and release of the inflammatory mediator IL-6 (Fig5C). Furthermore, RU486 treatment of S mice led to significantly increased expression of TNFR1 in liver and spleen (Fig5C). These data suggest that the TNF resistance of S mice depends entirely on GR biological activity. Because we previously found, using GR^dim^ mice which express a GR that is poor in dimerization, that protection by glucocorticoids (GCs) against TNF depends on GR dimerization and induction of GRE-element containing genes (Vandevyver *et al*, 2012b), we investigated whether the mouse *Mrc1* gene (of which miR-511 is spliced out), contains GRE elements in its promoter. To that end, we used the ConTra matrix (Hooghe *et al*, 2008) and found two GRE elements and one GR half binding site (Fig5D).

### miR-511 and TNFR1 are GC-regulated

**Figure 6:**
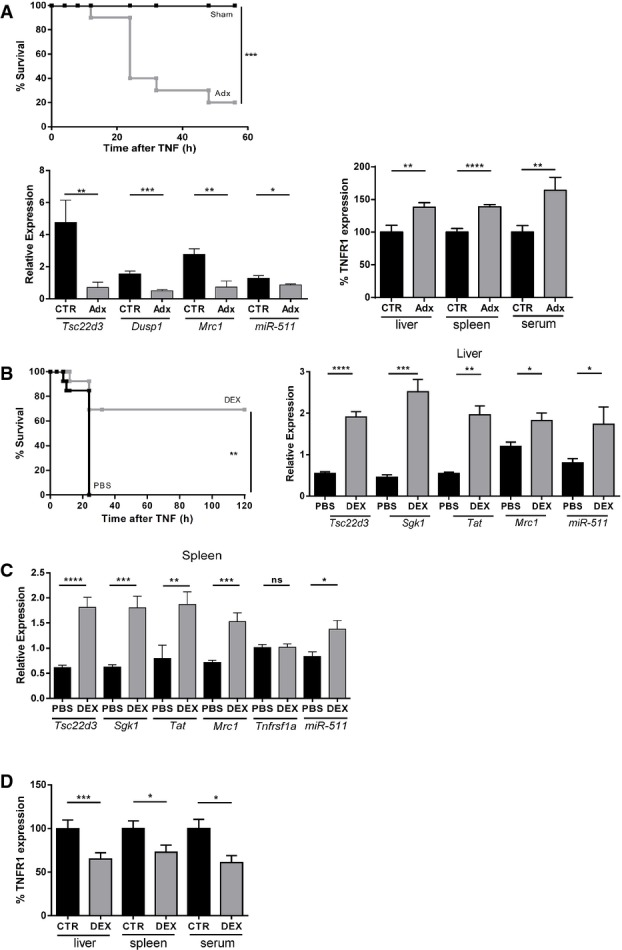
miR-511 expression depends on GCs Adx significantly sensitizes B mice for TNF. Sham-operated mice (black, *n *= 12) all resisted 2 μg TNF while most Adx mice (grey, *n *= 12) succumbed to this dose of TNF (top panel). Effect of adrenalectomy (Adx) on relative mRNA expression in the spleen of B mice (black, *n *= 5) and Adx mice (grey, *n *= 6). Bottom left panel shows significant down-regulation of *Tsc22d3* (encoding GILZ), *Dusp-1* (encoding MKP-1), *Mrc1* and miR-511 in Adx mice. Bottom right panel displays significant up-regulation of the protein level of TNFR1 in liver, spleen and serum of Adx mice (grey) compared to control mice (black) (all groups *n *= 10).

To study the dependency of expression of *Mrc1* and miR-511 as well as regulation of TNFR1 on GCs and GR, we adrenalectomized (Adx) mice to remove the major source of GCs. We confirmed that Adx significantly sensitizes mice for TNF-induced inflammation (Fig6A) (Bertini *et al*, 1988; Van Bogaert *et al*, 2011) and found that Adx decreased the expression of several validated GRE genes (*Tsc22d3*, encoding GILZ, and *Dusp1*, encoding MKP1) as well as *Mrc1* and miR-511 in the spleen and liver (Fig6A) and also strongly increased the expression of TNFR1 protein in these organs and in serum (Fig6A), supporting that Mrc-1 is a GRE gene and TNFR1 is under control of GCs.

Pretreatment with 50 μg DEX protects B mice against lethal SIRS induced by 30 μg TNF (−30 min, grey) compared to PBS pretreatment (250 μl, −30 min, black) (both groups *n *= 13) (left panel). Relative mRNA expression in the liver of B mice 6 h after i.p. injection of PBS (black, *n *= 10) or DEX (grey, *n *= 10) (right panel). Significant up-regulation of *Tsc22d3*, *Sgk*, *Tat* and *Mrc1*. Induction of miR-511 by DEX compared to PBS.

Relative mRNA expression in the spleen of B mice 6 h after i.p. injection of PBS (black, *n *= 10) or DEX (grey, *n *= 10). Significant up-regulation of *Tsc22d3*, *Sgk*, *Tat* and *Mrc1*. DEX had no effect on TNFR1 mRNA levels. Significant up-regulation of miR-511 by DEX.

Significant down-regulation of the protein level of TNFR1 in liver (*n *= 20), spleen (*n *= 20) and serum (*n *= 5) by 50 μg DEX (grey) compared to PBS (black). Data information: Data are presented as mean ± SE. Survival curves (Kaplan–Meyer plots) were compared by a log-rank test, and final outcomes were compared by a chi-square test. Differences between groups were assessed by Student’s *t*-test using unpaired comparisons and two-tailed analysis. *P < 0.05, **P < 0.01, ***P < 0.001, ****P < 0.0001.

Synthetic GCs such as dexamethasone (DEX) are frequently used as anti-inflammatory drugs, although there is still uncertainty about the mechanisms of action. A low dose of 50 μg DEX protected against TNF-induced lethal SIRS (Fig6B), and significantly induced the expression of several validated GRE genes, as well as *Mrc1* and miR-511 in liver and spleen (Fig6B and C). Expression of TNFR1 mRNA remained unchanged. In accordance with the induction of miR-511, TNFR1 protein expression decreased significantly after DEX in both organs and in the serum, suggesting that increased shedding of sTNFR1 is not involved in the reduced tissue expression of TNFR1 by DEX (Fig6D). These data demonstrate that Mrc-1 and miR-511 increase and TNFR1 protein decrease depend on GCs and GR and unfold a potential new mechanism of anti-inflammatory activity of these steroids, namely via miR-511 induction and TNFR1 reduction.

## Discussion

The mouse strain SPRET/Ei is derived from *Mus spretus*, which diverged some 1.5 million years ago from the house mouse, *Mus musculus*. The resulting genetic polymorphisms caused phenotypic differences that are relatively easy to map by linkage analysis. The SPRET/Ei (S) genome contains a SNP about every 100 bps compared to the genomes of standard *Mus musculus* strains such as C57BL/6J (B) (Dejager *et al*, 2009). S mice are extremely resistant to TNF-induced SIRS, a trait that is dominant and linked to proximal chr2 and distal chr6 (Staelens *et al*, 2002).

Because the *Tnfrsf1a* gene is centrally located in the critical region on chr6, we investigated the basis of the genetic linkage of TNF resistance to this gene. We found that S mice have 10 amino acid changes in TNFR1 compared to B mice, but the S TNFR1 receptor is fully functional when introduced in a B background. However, the TNFR1 protein is expressed at 2- to 5-fold lower level in S than in B mice, in serum and in all organs tested. Also, TNFR1 measured by FACS on splenic neutrophils confirmed the low TNFR1 membrane expression in S mice. The low serum levels of TNFR1 in S mice rule out that an extreme shedding of TNFR1 would for the basis of the low cell and tissue expression of TNFR1 in these mice. Thus, there is no qualitative but a quantitative link to TNFR1. In previous work, we have shown that decreased TNFR1 expression (50% in TNFR1^+/−^ mice) leads to complete resistance to TNF-induced SIRS in a non-linear gene-dosage way, meaning that 2× reduction in TNFR1 protein leads to increased LD_50_ of > 40× (Van Hauwermeiren *et al*, 2013) and this is linked with decreased TNF-induced intestinal barrier dysfunction (Van Hauwermeiren *et al*, 2014). We here report that proximal chr2 and distal chr6 are linked not only to the TNF resistance but also to the low TNFR1 protein levels of S mice, suggesting that the low TNFR1 levels are responsible for the resistance. For TNFR1 levels, the LOD scores of chr6 were far beyond highly significant, while the chr2 LOD score was only suggestive. With the poor breeding performance of interspecies crosses such as those between B and S mice, only small backcross populations could be obtained, leading to relatively broad peaks of linkage and big critical regions of several tens of cM. In favour of the data is the significant epistatic interaction between proximal chr2 and distal chr6 both at the level of resistance against TNF lethality (Staelens *et al*, 2002) and at the level of TNFR1 expression. Furthermore, chr6 consomic mice could be generated but not chr2 consomics because of sterility problems, which occur frequently in interspecies crosses (Dejager *et al*, 2009). The studies on chr6 consomic mice, however, led to the conclusion that regulation of the TNFR1 locus on chr6 does not depend only on the locus itself, but possibly also on the chr2 locus. Because the weak expression of TNFR1 in S mice was observed only on the protein level and not the mRNA level, its regulation by miRs was an interesting hypothesis, especially since the regulation of TNFR1 remains very poorly documented.

Over the past decade, miRs have emerged as important regulators of mRNA stability and translation (Hausser & Zavolan, 2014). Hundreds of miRs and their possible targets have been discovered in the genomes of plants and animals (Bartel & Chen, 2004; Valencia-Sanchez *et al*, 2006). Some miRs are essential as negative-feedback molecules in inflammation and their anti-inflammatory effects have been validated, such as miR-155 and miR-146a (Tsitsiou & Lindsay, 2009). Most miRs bind to 3′ UTRs of regulated mRNAs and lead to faster degradation of mRNA, but many miRs can leave the mRNA levels intact and inhibit translation. An example of the latter is miR-579 (Han *et al*, 2013). We found that miR-511 was repeatedly predicted as a TNFR1 regulator by several algorithms, that it is the only putative TNFR1 regulator located in the critical region of chr2 and that it is expressed at significantly higher levels in S than in B mice. miR-511 is located in an intron of the *Mrc1* gene and was suggested to be regulated by the promoter of the host gene (Squadrito *et al*, 2012). The *Mrc1* gene is expressed predominantly by macrophages (Squadrito *et al*, 2012), but also by liver, lymph nodes and spleen (Supplementary Fig S5). In agreement with *Mrc1*/miR-511 co-regulation, we found higher *Mrc1* mRNA levels in S than B liver and spleen. We also found that miR-511 indeed regulates the TNFR1 3′ UTR in a reporter system, that transfection of this miR down-regulates TNFR1 protein in B and S primary fibroblasts moderately and that anti-miRs have the inverse effect.

Interestingly, transient *in vivo* delivery of miR-511 down-regulated TNFR1 in livers of mice and protected them not only against TNF, but also against endotoxic shock (in a TNFR1-dependent way) and in a model of TNF-mediated lethal hepatitis (induced by ConA). The experimental delivery system of the miRs and anti-miRs targets only the liver of mice (Liu *et al*, 1999), and hence the effect of liver-specific regulation of TNFR1 on the lethal response to TNF is interesting. Remarkably, a modest decline in TNFR1 protein expression by miR-511 was able to protect the mice against TNF. This is in line with the extreme non-linear gene-dosage effect we described recently (Van Hauwermeiren *et al*, 2013). Also, the liver-specific down-regulation of TNFR1 via hydrodynamic tail injection suffices to protect against TNF-induced lethal shock, suggesting that liver TNFR1 plays an important role, and this is in line with our previous findings (Van Bogaert *et al*, 2011). Finally, we opted for the ConA model and not for the well-known TNF + galactosamine model, because in the latter model, only weak effects of partial TNFR1 concentration changes were found (Van Hauwermeiren *et al*, 2013). Our findings indicate miR-511 is a genuine TNFR1 regulator and has therapeutic potential, at least in mice. Optimization of therapy with miR-511 by more general delivery and safety of this therapy should be addressed in the future. Furthermore, the miR-511-TNFR1 axis has yet to be validated in humans. In Supplementary Fig S6, we display the high sequence similarity of the mouse (C57BL6) and human miR-511 seed sequence and two TNFR1 target sequences. Also, anti-miRs inhibiting miR-511 delivered specifically in the liver of mice increased TNFR1 expression and sensitized them for TNF-induced SIRS. Since also S mice were sensitized by this anti-miR delivery, we propose that at least part of the extremely robust TNF resistance of S mice depends on miR-511. Final proof that miR-511 is responsible for part of the TNF resistance and low TNFR1 protein levels of S mice should come from genetic disruption of this miR-511, but genetic manipulation of S mice has not been possible so far despite intense efforts (Hochepied *et al*, 2004). Finally, *in silico* prediction of other targets of miR-511, using miR Walk, revealed no less than 997 targets which are predicted by at least 4/10 programs, including some that are important players in the TNF and LPS signalling pathways, that is TRAF2 (Supplementary Fig S2B).

The genetic link of TNF resistance and low TNFR1 levels to chr2 and chr6 is difficult to explain. We found only minimal changes in the miR-511 target sequence between B and S in the *Tnfrsf1a* 3′ UTR, and both these sequences responded equally well to transfected miR-511. One possibility is that subtle differences in the entire TNFR1 mRNA sequence may cause better response of the S *Tnfrsf1a* gene to miR-511 because of different 3D mRNA structure. Furthermore, it is not easy to explain the linkage to the miR-511 locus if the sequence of miR-511 appears to be identical in B and S mice. It is clear that we observe higher expression of *Mrc1* and miR-511 in S than in B tissues. We previously found that S mice have an overactive HPA, which is stimulated by stress and leads to ACTH release from the pituitary and corticosterone (CS) release from adrenals. In S mice, high CS levels lead to high basal expression levels of GR-induced genes (Dejager *et al*, 2010). GR can homodimerize and act as a true transcription factor, binding to GRE-like elements and strongly inducing gene expression. The canonical GRE element resembles AGAACA(N)_3_TGTTCT. Such elements can be found near transcription start sites of GRE genes (Wiench *et al*, 2011). Typical GRE genes are involved in gluconeogenesis (*Sgk*, *Tat*) or have anti-inflammatory effects (*Tsc22d3*, *Dusp1*). Using GR^dim^ mice, which express a mutant GR that is poor in dimerization, it was recently shown that GR dimerization followed by GRE-gene induction is essential in its protection against SIRS (Kleiman *et al*, 2012; Vandevyver *et al*, 2012b). Interestingly, inhibition of GR by the irreversible inhibitor RU486 sensitized both B and S mice to TNF, but also led to complete loss of differences in TNF response be tween B and S, which means that the spectacular TNF resistance of S mice totally depends on their overactive HPA. We believe that our data indicate that both phenotypes (low TNFR1 protein and TNF resistance) depend on the HPA but are linked with a downstream GRE gene, relevant in this model and that mediates the anti-inflammatory effect of GR, and is located on proximal chr2, namely the *Mrc1*/miR-511 locus. We indeed found that: (i) treatment of S mice with RU486 led to increased TNFR1 protein levels and increased TNF sensitivity, (ii) removal of adrenals in mice markedly reduced the basal expression levels of *Mrc1* and miR-511 and increased TNFR1 levels and TNF sensitivity, (iii) injection of DEX (a synthetic GC) in mice led to induction of these genes and to reduction of TNFR1 protein expression in tissues and in the serum (thus in a shedding independent way), (iv) that the induction of miR-511 and reduction of TNFR1 protein is not observed in GR^dim^ mice (data not shown) and (v) in the promoter of the *Mrc1* gene, we found GRE elements, and *Mrc1* was proven to be induced by GCs (Ehrchen *et al*, 2007). GCs have been shown to acutely inhibit miR processing enzymes such as Drosha and Dicer (Smith *et al*, 2010), but also to induce miRs in several cell systems (Izzotti *et al*, 2010; Smith *et al*, 2013). Our data suggest that in S mice, an overactive HPA leads to high expression of miR-511 and hence strong down-regulation of TNFR1 protein expression and less response to TNF. Hence, GCs, either endogenous or therapeutically administered, are clearly able to induce miRs that could inhibit pro-inflammatory targets.

A new regulatory network important in TNF biology is appearing from our studies. We show that miR-511 is strongly induced by GCs and is a true TNFR1 modulator and thus an anti-inflammatory miR. Since minimal reductions of TNFR1 have considerable effects on TNF sensitivity (Van Hauwermeiren *et al*, 2014), we believe that at least part of the anti-inflammatory effects of GCs are mediated by induction of this miR, resulting in reduced TNFR1 expression. Further studies are needed to determine whether this anti-inflammatory system encompasses other inflammatory mediators and targets.

## Materials and Methods

### Mice

C57BL/6J (B) mice were purchased from Janvier-Europe. SPRET/Ei (S) mice were obtained from The Jackson Laboratory and bred in our facility. (BXS)F1 mice were generated by crossing female B mice with male S mice. B.S.chr6 consomic mice were generated by backcrossing (BxS)F1 mice with the host strain, B, and then repeatedly backcrossing to the host strain and screening the progeny for the non-recombined S donor locus of interest (*Tnfrsf1a*) in each generation (Nadeau *et al*, 2000). B.S.chr6 consomic mice heterozygous for the S *Tnfrsf1a* allele were intercrossed at the N10 generation, and resulting N10F2 mice homozygous for the B *Tnfrsf1a* allele and mice homozygous for the S *Tnfrsf1a* allele and heterozygotes were identified by typing for the polymorphic markers around the *Tnfrsf1a* gene. TNFR1^−/−^ mice generated by Dr. M. Rothe (Rothe *et al*, 1993) were a kind gift from Dr. H. Bleuthmann. TNFR1^+/−^ mice were generated by crossing TNFR1^−/−^ mice with B mice. Adrenalectomized B mice were purchased from Janvier-Europe and received 0.9% NaCl in their drinking water. GRdim mice and the WT controls had an FVB background and were described before (Vandevyver *et al*, 2012b). All mice were kept in individually ventilated cages under a constant dark–light cycle in a conventional animal house and received food and water *ad libitum*. The mice were used at the age of 8–12 weeks and were all female. Animal experiments were approved by the institutional ethics committee for animal welfare of the Faculty of Sciences, Ghent University, Belgium. Genome sequences of SPRET/Ei were retrieved from the Sanger Laboratories ftp://ftp-mouse.sanger.ac.uk/current_snps/mgp.v3.snps.rsIDdbSNPv137.vcf.gz.

### Compounds, injections and measurements

Recombinant mouse TNF (specific activity of 1.66 × 10^9^ IU/mg) was expressed in *Escherichia coli* and purified in our laboratory. The preparation contained less than 6 EU/mg protein of endotoxin as determined by a *Limulus* amoebocyte lysate assay and had a concentration of 1 mg/ml. Lipopolysaccharide (LPS) from *Salmonella abortus equii*, RU486 and ConA were purchased from Sigma. RU486 was dissolved in DMSO and 5 mg was injected in 50 μl DMSO per mouse. DMSO was used as solvent control. Dexamethasone was bought as a ready-to-inject solution called Rapidexon from Medini N.V. and injected i.p. at 50 μg/mouse. On one occasion, for the induction of *Mrc1* in B mice, 500 μg Rapidexon was injected. Mice were injected intraperitoneally with TNF or LPS in 0.3 ml of pyrogen-free phosphate-buffered saline (PBS). Mortality was recorded regularly until no further deaths occurred. Rectal body temperatures were measured with an electronic thermometer from Comark. Concanavalin A (360 μg in 200 μl per mouse) was injected intravenously. Two hours later, 100 μl of blood was withdrawn from the retro-orbital plexus for serum TNF detection. Six hours later, another blood sample was taken, and serum alanine aminotransferase (ALT) was measured using a Hitachi kit and apparatus in the Clinical Biology Laboratory of Ghent University Hospital. The bio-activity of TNF in the serum was measured with an MTT cell death assay using the sensitive L929 cell line. Serum IL-6 was measured using a specific bio-assay based on the proliferating effect of IL-6 on 7TD1 hybridoma cells.

### *Tnfrsf1a* 3′ UTR reporter construct and luciferase reporter assay

The DNA coding for the *Tnfrsf1a* 3′ UTR of B and S was purchased from Genscript. The 514-bp sequence of B and the 508-bp sequence of S were digested out of the vector pUC57 with SgfI and NotI and ligated in the psiCHECK-2 vector (Promega). The constructs were transformed into MC1061 cells, and transformants were screened for the presence of a correct sequence by using PCR primers CAGATGAAATGGGTAAGTAC and AAACCCTAACCACCGCTTAA. The 3′ UTR reporter plasmids were co-transfected with pre-miR precursor molecules (Supplementary Fig S4) in HEK-293T cells using Lipofectamine 2000 (Invitrogen). Cells were harvested 48 h post-transfection, washed with PBS and lysed in passive lysis buffer (PLB). Firefly luciferase activity was measured in Optiplate-96 F plates (Perkin Elmer) by adding Luciferase Assay Reagent II (Promega) to generate a luminescent signal. This signal was quantified using the GLOMAX 96 microplate luminometer (Promega). Afterwards, this reaction was quenched, and the Renilla luciferase reaction was initiated by adding Stop & Glo® Reagent (Promega) to the same wells.

### Hydrodynamic injection in mice

A precursor miRNA expression clone for mmu-miR-511 (GAUACCCACCAUGCCUUUUGCUCUGCACUCAGUAAAUAAUAAUUUGUGAAUGUGUAGCAAAAGACAGGAUGGGGAUCCA), cloned in the pEZX-MR04 vector, was purchased from GeneCopoeia, as well as a scrambled control clone (Supplementary Fig S4). LNA-enhanced miRCURY i-mmu-miR-511-5p (GAGTGCAGAGCAAAAGGCA) and i-miR-511-5pMMControl were purchased from Exiqon (Supplementary Fig S4). Plasmid DNA was dissolved in PBS, and 10 μg in a volume of 2 ml was injected in the tail vein under high pressure. This technique guarantees hepatocyte-specific uptake and transient expression of the plasmid (Liu *et al*, 1999).

### mTNFR1 ELISA and CS ELISA

Liver, kidney, lung and spleen of mice were excised and snap-frozen in liquid nitrogen. Samples were homogenized in PBS containing 0.5% CHAPS and complete protease inhibitor cocktail tablets from Roche. Homogenates were centrifuged for 30 min at 20,000 *g* and 4°C, after which the supernatant was collected and stored at −80°C. Blood was collected by retro-orbital bleeding and allowed to clot for 1 h at 37°C. Serum was prepared and stored at −20°C. Protein concentration was determined by the Bradford method (Bio-Rad), and 500 μg was used to perform an ELISA specific for TNFR1 using the mouse sTNF RI/TNFRSF1A duoset ELISA from R&D Systems. The levels were normalized to the levels of C57BL/6 or control samples, which were set as 100%.

CS was measured in serum using an ELISA (ARBOR Assays) as suggested by the manufacturer.

### FACS measurement of TNFR1 on neutrophils of mice

Spleens were harvested from untreated wild-type, TNFR1 KO and SPRET mice, and single cells were obtained by teasing the spleen apart by pressing it through a 100-μm cell strainer, eliminating clumps and debris. Cells were collected in 10 ml DPBS and were spin down (4°C, 7 min, 400× *g*). Cells were lysed with 1 ml of lysis buffer (8.3 g NH_4_Cl, 1 g KHCO_3_ and 200 μl 0.5 mol/l EDTA), washed and counted. A total of 1 × 10^6^ splenocytes were stained for 30 min at 4°C in the dark with the following anti-mouse antibodies: anti-TCRb-FITC, anti-CD19-PECy7, anti-CD11b-Pacific Blue, anti-Ly6C-APC, anti-Ly6G-AF700 (all from BD Biosciences) and anti-TNFR1-PE (BioLegend). Cells were washed and dissolved in FACS buffer. Fluorescent events were acquired using an LSR2 and analysed using FACSDiva software (BD Biosciences). Neutrophils were defined as SSC^high^CD11b^+^Ly6G^high^Ly6C^high^. After gating neutrophils (see Supplementary Fig S7), TNFR1^+^ cells were plotted in a histogram. The correct neutrophil population and TNFR1^+^ populations were gated using an FMO conditions without anti-Ly6G-AF700 and anti-TNFR1-PE antibodies, respectively. Analysis was performed using FlowJo software (Tree Star, Inc.)

### qPCR

Liver, kidney, lung and spleen samples were stored in RNA later® from Ambion. Samples were homogenized and RNA was extracted using an RNeasy mini kit from Qiagen. RNA concentration was measured with the NanoDrop1000 from ThermoScientific, and 500 ng RNA was used to prepare cDNA with iScript from Bio-Rad. qPCR was performed using the SYBR Green master mix and the LightCycler 480 from Roche with the following primers: 5′-CCGGGAGAAGAGGGATAGCTT-3′ and 5′-TCGGACAGTCACTCACCAAGT-3′ for mTNFR1, 5′-GCTGAATCCCAGAAATTCCGC-3′ and 5′-ATCACAGGCATACAGGGTGAC-3′ for Mrc1, 53′ and 5′-TCGGACAGTCACTCACCAAGT-3′ for mTNFR1, 5′-GCTGAATCCCAGAAATTCGilz, 5z, 5 5 5z, 5, 5 CGGACAGTCA and 5 and 5, 5 CGGACAGTCACTCACCA for CGC-3 and 5 and 5, 5 CGGACAGTCACTCAC for Mrc1, 5′-TGAAGCAGGCATCTGAGGG-3′ and 5′-CGAAGGTGGAAGAGTGGGAG-3′ for Gapdh, and 5′-CCTGCTGCTCTCAAGGTT-3′ and 5′-TGGCTGTCACTGCCTGGTACTT-3′ for Rpl13a. The best performing housekeeping genes were determined with geNorm (Vandesompele *et al*, 2002). No SNPs between C57BL/6 and SPRET/Ei were included in the primers (not shown). qPCR for miR-511 was done using specific MultiScribe™ cDNA synthesis and TaqMan® Pri-miRNA Assays for mmu-miR-511 and three stable miRs (mmu-miR-194, mmu-miR-24 and mmu-miR-29a from Applied Biosystems). All values shown are relative expression values normalized to the geometric mean of the selected housekeeping genes.

### Quantitative trait loci (QTL) mapping

To map the loci responsible for low TNFR1 protein levels in S mice, an interspecies backcross between female (C57BL/6 × SPRET/Ei) F1 mice and male C57BL/6 mice was set up and N2 backcross mice were generated. Tail biopsies were collected at weaning from 214 N2 mice, and high-quality genomic DNA was prepared by standard phenol–chloroform extraction. A genome scan on 100 ng DNA was performed with 72 microsatellite markers. Primer sequences from the Massachusetts Institute of Technology (MIT) were retrieved from www.informatics.jax.org. Coverage of the genome was estimated by taking the position of the marker loci on the Mouse Genome Database genetic map obtained from The Jackson Laboratory and applying a swept radius of 20 cM (Silver, 1995). Livers from the 214 N2 backcross mice were excised at the age of 8 weeks and snap-frozen in liquid nitrogen. Total protein was isolated and 500 μg was used to measure TNFR1 levels by ELISA. After the first screening, the density of markers was increased on chromosomes 2 and 6, which were shown to be linked to the trait. Linkage analysis was performed using the R/qtl software version 1.12-26 running under R 2.9.1 (Broman, 2003). Significance thresholds of LOD scores were estimated by 10,000 permutations of experimental data. For TNF resistance, the 5% significance thres-hold LOD score is 2.50, the 10% threshold is 2, and the suggestive level is 1.4. For TNFR1 protein levels, the 5% significance threshold LOD score is 2.41, the 10% threshold is 2.12, and the suggestive threshold is 1.34. The protein level was analysed using a normal model by the EM algorithm in R/qtl (Lander & Botstein, 1989).

### Mouse embryonic fibroblast (MEF) studies

MEFs were isolated from embryos 18 days post-coitum. They were cultured in DMEM medium (supplemented with 10% FCS, penicillin, streptomycin, sodium pyruvate and L-glutamine) and seeded in culture flasks. Cells were trypsinized and seeded in 24-well plates at 100,000 cells per well. Pre-miR™ miRNA Precursors for mmu-mir-511 (GAUACCCACCAUGCCUUUUGCUCUGCACUCAGUAAAUAAUAAUUUGUGAAUGUGUAGCAAAAGACAGGAUGGGGAUCCA) and a negative control were purchased from Ambion (Supplementary Fig S4). LNA-enhanced miRCURY i-mmu-miR-511-5p (GAGTGCAGAGCAAAAGGCA) and i-miR-511-5pMMControl were purchased from Exiqon. Fifty micromoles of the molecules was transfected using Lipofectamine RNAiMAX from Invitrogen, and 24 h later the cells were lysed with PBS containing 0.5% CHAPS and complete protease inhibitor cocktail tablets from Roche. TNFR1 was measured using the TNFR1 ELISA.

### ConTra analysis of *Mrc1* promoter

Analysis of the 2000-bp upstream *Mrc1* promoter for GRE sequences across species was conducted using the ConTra online software (Hooghe *et al*, 2008), with the highest stringency of core = 1.00 and similarity matrix = 0.95 and a method to minimize false positives with TRANSFAC matrices. (http://bioit.dmbr.ugent.be/contrav2/cite_newstyle.php).

### Statistical analysis

Survival curves (Kaplan–Meyer plots) were compared by a log-rank test, and final outcomes were compared by a chi-square test. Data are expressed as the means ± SE. Statistical significance of differences between groups was evaluated with Student’s *t*-tests using unpaired comparisons and two-tailed analysis. Error bars in the figures represent the mean ± SE *, **, *** and **** represent *P* < 0.05, *P* < 0.01, *P* < 0.001 and *P* < 0.0001, respectively. The relevant, exact *P*-values are summarized in Supplementary Fig S8.

**The paper explained**

**Problem**

Sepsis is an acute inflammatory condition resulting from infection. It is a huge unmet medical need and highly lethal. Worldwide at least 20 million people suffer from sepsis each year. Several key molecules in sepsis have been identified. One of them is tumour necrosis factor (TNF). But numerous clinical trials inhibiting TNF have not resulted in a solid new therapy based on TNF inhibition.

**Results**

We believe that the major TNF receptor, TNFR1, which is ubiquitously expressed, is a good and more specific therapeutic target for sepsis. We have found a mouse strain, SPRET/Ei, which is very resistant to TNF-induced acute, lethal inflammation. This trait appears to result from a very low expression of the TNFR1 protein, and this in turn is under the control of a microRNA, namely miR-511. We validate miR-511 as a true TNFR1-regulating miR, which can be delivered to mice and then reduces the TNFR1 levels and protects mice in the TNF and other mouse inflammation models. Furthermore, this miR-511 is strongly induced by glucocorticoids, which are the standard first-line anti-inflammation therapeutics. Thus, our work also uncovers a novel anti-inflammatory pathway of glucocorticoids.

**Impact**

Although therapeutic use of microRNAs is a hotly debated issue, our data show that microRNA delivery to a mammalian organism (mouse) can down-regulate the TNFR1 protein expression. Whether that result can also be translated to humans is not yet known, and whether that effect has off-target effects is also unknown so far. But the data underline that TNFR1 is an interesting drug target in acute inflammation.

## Supporting Information

**Supplementary Figures**

**Review Process File**
