# Supplementary material for: Glucocorticoid-induced microRNA-511 protects against TNF by down-regulating TNFR1
Source: EMBO Mol Med. 2015 May 20;7(8):1004–17. doi: 10.15252/emmm.201405010 (PMC4551340; doi:10.15252/emmm.201405010)
Supplement: Supplementary file 1 [file emmm0007-1004-sd1.pdf]

Supplemental data Contents

Legends to the supplemental figures S1, S2A, S2B, S3, S4, S5, S6, S7, S8

Supplemental Fig. S1

Supplemental Fig. S2A

Supplemental Fig. S2B

Supplemental Fig. S3

Supplemental Fig. S4

Supplemental Fig. S5

Supplemental Fig. S6

Supplemental Fig. S7

Supplemental Fig. S8

Fig. S1: The table shows amino acid differences in the TNFR1 protein between SPRET/Ei, DBA2 and C57BL6/J mice as well as other species. The figure represents the different domains of TNFR1 and the amino acid differences between C57BL6/J and SPRET/Ei. (PLAD: pre-ligand association domain; CRD: cystein rich domain; CTD: cytoplasmic topological domain; DD: death domain).

Fig. S2A: Table representing miRs, their chromosomal location and their prediction to have TNFR1 3'UTR as a target by different on-line available algorithms. Based on the Ensembl (www.ensembl.org) annotation, only one protein coding Tnfrsf1a transcript, the consensus CDS, has a complete 3'UTR. One more transcript with a 3'UTR is annotated as nonsense mediated decay and not taken into account. So only the transcript Tnfrsf1a-001 (ENSMUST00000032491) has to be, and has been, screened for miR-511 target site(s).

Fig. S2B: Selection of other miR-511 targets predicted by at least 4/10 of the prediction programs used in Fig. S2A. On the left: target genes which are predicted by Ingenuity Pathway Analysis to be linked to TNF induction and response. On the right: target genes which are predicted by Ingenuity Pathway Analysis to be linked to LPS response. There are many (n=997) targets, besides *Tnfrsf1a* mRNA, predicted to be regulated by miR-511 by the 10 prediction programs of Fig S2A. There are some interesting potential targets that may contribute to the anti-inflammatory effects of miR-511, besides TNFR1 regulation, e.g. TRAF2.

Fig. S3: *In vivo* effect 24 h after hydrodynamic injection of (BxS)F1 mice with plasmids expressing anti-miR-511 (grey, n=9), anti-miR-CTR (n=10) or PBS (n=10). TNFR1 protein levels in the liver were measured by ELISA 24 h after hydrodynamic injection (left panel). Survival of (BxS)F1 mice injected with 500 μg TNF 24 h after hydrodynamic injection (middle panel). Mice pretreated with anti-miR-511 (grey, n=9) were significantly sensitized for TNF compared to the PBS control group (●, black, n=10) and showed the largest drop in body temperature (right panel) of all groups.

Fig. S4: Table representing the miR and anti-miR tools used *in vitro* and *in vivo*.

Fig. S5: Microarray data analysis of *Mrc1* expression via the toolbox Genevestigator (Hruz et al., 2008). The top 30 anatomical parts (from 182 in total) in which *Mrc1* is expressed are displayed, based on 4675 Affymetrix 430 2.0 microarrays. The level of expression within a tissue type is the average expression across all samples that were annotated with that particular tissue type and the whiskers indicate the standard error of the mean. The number of samples that were included to calculate this average is indicated on the right of the graph.

Fig. S6: Top: A comparison of the mouse (C57BL/6, B) and human (H) miR-511 binding sites in the 3' UTR of the *Tnfrsf1a* resp. *TNFRSF1A* genes which are 17/20 identical (most 5' target sequence shown in Fig. 3B) and 7/19 identical (most 3' target sequence of Fig. 3B). Thus the 5' most target sequence is very similar in mouse and human. Middle: the miR-511 seed sequence of 21 nucleotides of mouse (B) and human (H) are 86% identical, and differ only at three nucleotides. Bottom: an alignment of human miR-511 and both target sequences, showing that in the human case, the miR-511 hybridizes with 12/21 nucleotides to the first target sequence, versus 15/21 in mouse. The experimental validation of the miR-511-TNFR1 axis has to be done before extrapolation to the human system is possible.

Fig. S7: Flow cytometric (FACS) analysis of neutrophils in spleens of untreated wild type, TNFR1 KO and SPRET mice. Neutrophils were defined as $SSC^{high}CD11b^{+}Ly6G^{high}Ly6C^{mid}$. After gating neutrophils, $TNFR1^{+}$ cells were plotted in a histogram.

Fig. S8: Table of the exact p-values of statistical tests, displayed on the figures.

S1

position	SPRET/Ei	DBA/2	C57BL/6	Danio rerio	Gallus gallus	Mus musculus	Rattus norvegicus	Bos taurus	Canis lupus	Macaca mulatta	Homo sapiens	Pan troglodytes	domain
36	N	D	D	G	R	D	D	D	N	-	D	D	PLAD
135	H	Y	Y	K	F	Y	Y	Y	Y	Y	Y	Y	CRD 3
167	T	N	N	K	R	N	N	H	T	T	T	T	CRD 4
189	Q	E	E	-	E	E	Q	K	T	L	L	L	CRD 4
242	S	P	P	K	S	P	P	P	P	S	S	S	CTD
252	S	P	P	S	P	P	S	S	P	S	S	S	CTD
291	P	S	S	-	-	S	H	-	-	-	-	-	CTD
308	Y	H	H	-	-	H	H	S	P	P	P		CTD
394	H	R	R	R	-	G	G	G	G	G	G	G	DD
431	T	A	A	V	S	A	R	L	L	L	L	L	DD

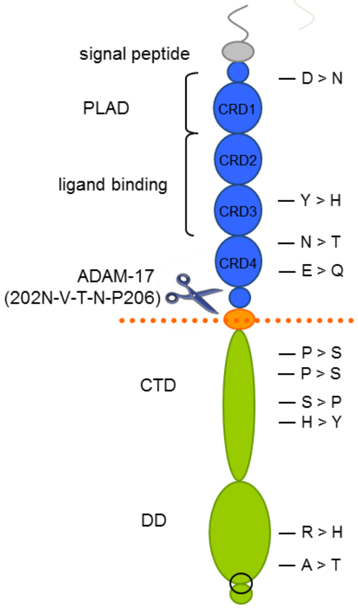

S2A

	chromosome	DIANAmT	miRanda	miRDB	miRWalk	RNAhybrid	PICTAR4	PICTAR5	PITA	RNA22	Targetscan	SUM
**mmu-miR-29a/b1**	6	1	1	0	1	1	0	1	0	0	1	**6**
**mmu-miR-29b2**	1	1	1	0	1	1	0	1	0	0	1	**6**
**mmu-miR-125a-5p**	17	1	1	0	1	0	0	1	0	0	1	**5**
**mmu-miR-125a-3p**	17	1	0	0	0	0	0	0	0	0	0	**1**
**mmu-miR-125b**	9	0	1	0	1	0	0	1	0	0	1	**4**
**mmu-miR-128-1**	1	0	1	0	0	0	0	0	0	0	0	**1**
**mmu-miR-138-1**	9	0	0	0	0	0	0	1	0	0	0	**1**
**mmu-miR-142**	11	0	1	0	0	0	0	0	0	0	0	**1**
**mmu-miR-149**	1	1	1	0	1	0	0	1	0	0	1	**5**
**mmu-miR-181c**	8	0	1	0	0	0	0	0	0	0	0	**1**
**mmu-miR-211**	7	1	0	0	0	0	0	1	0	0	0	**2**
**mmu-miR-296**	2	0	1	0	0	0	0	1	0	0	1	**3**
**mmu-miR-335**	6	0	1	0	0	0	0	1	0	0	0	**2**
**mmu-miR-351**	X	1	1	0	1	0	0	1	0	0	1	**5**
**mmu-miR-511**	2	0	1	0	1	0	0	1	0	0	1	**4**
**mmu-miR-592**	6	0	0	0	0	0	0	1	0	0	0	**1**
**mmu-miR-680-1**	6	1	1	0	1	0	0	0	0	0	1	**4**
**mmu-miR-763**	10	1	1	0	1	0	0	0	0	0	1	**4**

S2B

Abca1	Ednrb	Met	Slc14a1
Abcb1b	Efnb2	Msx2	Slc5a8
Abcd2	Ehf	Mylk	Slc7a8
Acaca	Elavl1	Ncf2	Slc8a1
Adora2a	Esr1	Nfkbie	Socs2
Adrb1	Fbxo32	Nr0b2	Sp1
Alox8	Fgf10	Olr1	Star
Angpt2	Gab1	Pappa	Stat5a
Apln	Gadd45g	Pde4b	Steap4
Aqp1	Gria1	Pemt	Tapbp
Aqp3	Ier3	Plagl2	Tgfbr1
Aqp9	Il17rd	Ppara	Timp3
Arc	Itgam	Ppp2r1b	Tnfrsf1a
Bace1	Lep	Psen1	Tnfsf10
Bcl2l2	Lrp6	Ptgfr	Traf2
Bmpr1a	Ltbr	Ptgs2	Tub
Cd3e	Man1a2	Rassf7	Twist1
Ciita	Man1c1	Scn9a	Ucp3
Clec5a	Map2k4	Scnn1a	Vcl
Cpt1a	Mapkap1	Scube2	Vdr
Crem	Mat2a	Sdc2	
Csf1	Mbp	Sele	
Cxcr5	Mc1r	Serpind1	
Cyp7a1	Mecp2	Slc12a6	

Traf2	Mdfic
Tollip	Invs
Tnfsf10	Iigp1
Tnfrsf1a	Gpnmb
Slc14a1	Gbp3
Sele	Gadd45g
Sarm1	Fgl2
Rsad2	Esr1
Rhbdf2	Cyp7a1
Ptgs2	Clec5a
Ppara	Cldn1
Plek	Aqp1
Parp9	

S3

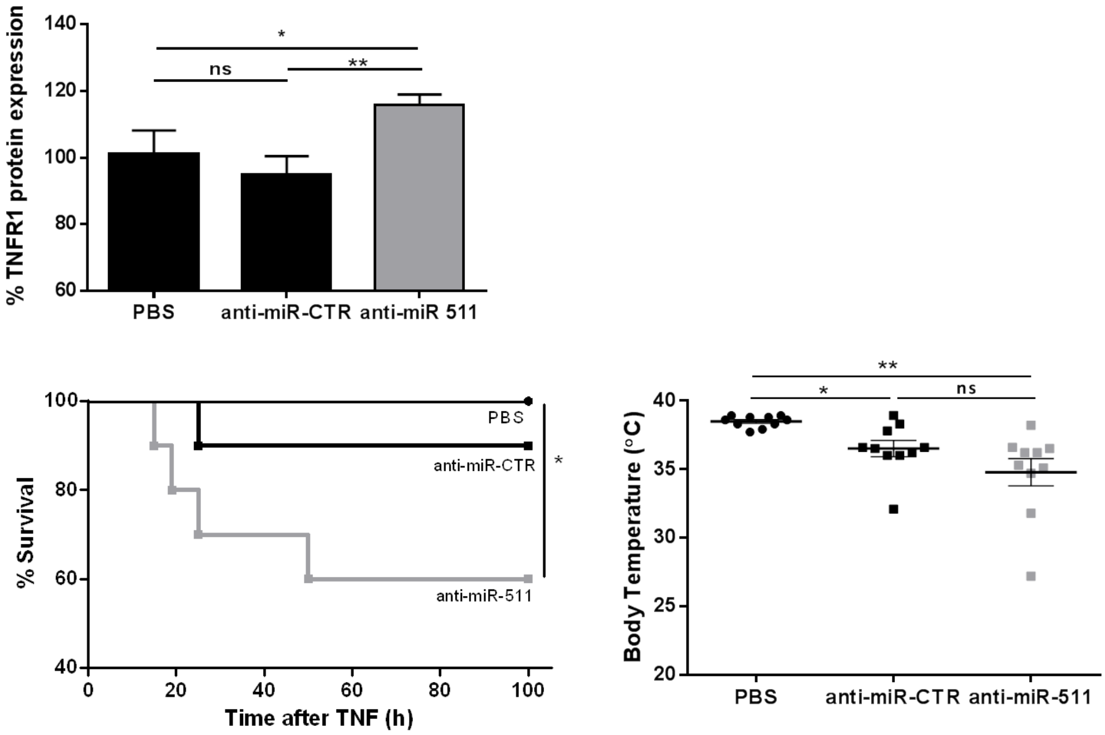
% TNFR1 protein expression
140
120
100
80
60
*
ns
**
PBS
anti-miR-CTR
anti-miR 511
% Survival
100
80
60
40
0
20
40
60
80
100
Time after TNF (h)
PBS
anti-miR-CTR
anti-miR-511
*
Body Temperature (°C)
40
35
30
25
20
**
*
ns
PBS
anti-miR-CTR
anti-miR-511

S4

	in vitro	*in vivo*
miR	premiR precursor molecule 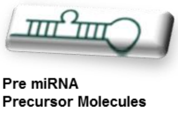	premiR precursor expression clone 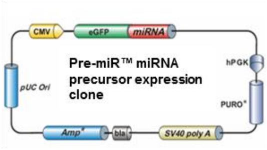
anti miR	LNA miR inhibitory molecule 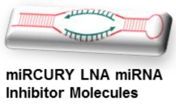	LNA miR inhibitory molecule 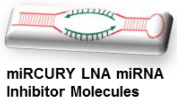

S5

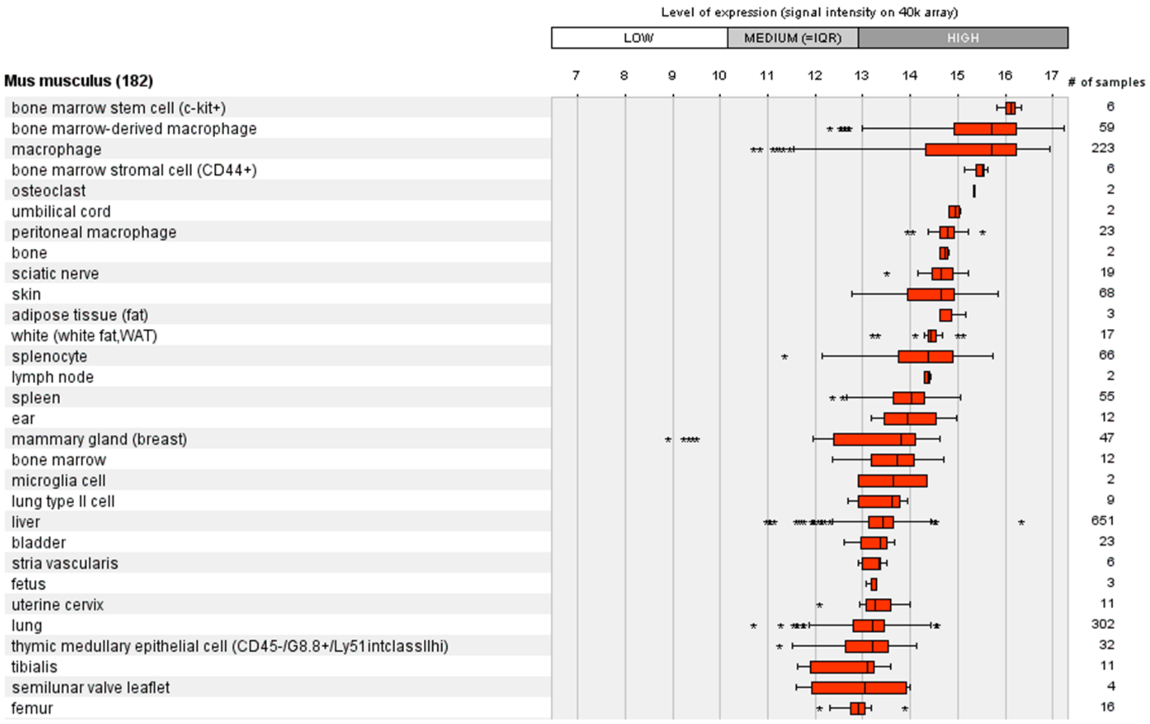
Level of expression (signal intensity on 40k array)
LOW
MEDIUM (=IQR)
HIGH
Mus musculus (182)
7 8 9 10 11 12 13 14 15 16 17
# of samples
bone marrow stem cell (c-kit+) 6
bone marrow-derived macrophage 59
macrophage 223
bone marrow stromal cell (CD44+) 6
osteoclast 2
umbilical cord 2
peritoneal macrophage 23
bone 2
sciatic nerve 19
skin 68
adipose tissue (fat) 3
white (white fat,WAT) 17
splenocyte 66
lymph node 2
spleen 55
ear 12
mammary gland (breast) 47
bone marrow 12
microglia cell 2
lung type II cell 9
liver 651
bladder 23
stria vascularis 6
fetus 3
uterine cervix 11
lung 302
thymic medullary epithelial cell (CD45-/G8.8+/Ly51intclassIIhi) 32
tibialis 11
semilunar valve leaflet 4
femur 16

S6

Target sequence 1

```
mRNA B 5' G G A T T G T A G A G G A A A G G C A C 3'
          : :   : : :     : : : : : : : : : : : :
mRNA H 5' G G T T T G C - G A G G A G A G G G A C 3'
```

Target sequence 2

```
mRNA B 5' G A C A A G C A T A G - - A A A G G C G G 3'
          :         :       :       : :         : :
mRNA H 5' G G G G C G A G C A C G G A A C A A T G G 3'
```

miR-511 seed sequence

```
miR511 B 3' A C U C A C G U C U C G U U U U C C G U A 5'
            : : :   : : : : : : : : : : : : :   : : :
miR511 H 3' A C U G A C G U C U C G U U U U C U G U G 5'
```

Target sequence 1 aligned with miR-511 sequence

```
mRNA   5' G G T T T G C - G A G - G A G A G G G A C 3'
            :     : : :   : : :     :   : :       : :
miR511 3' A C U G A C G U C U C G U U U U C U G U G 5'
```

Target sequence 2 aligned with miR-511 sequence

```
mRNA   5' G G G G C G A G C A C G G A A C A A T G G 3'
            :       :       :       : :     :
miR511 3' A C U G A C G U C U C G U U U U C U G U G 5'
```

S7

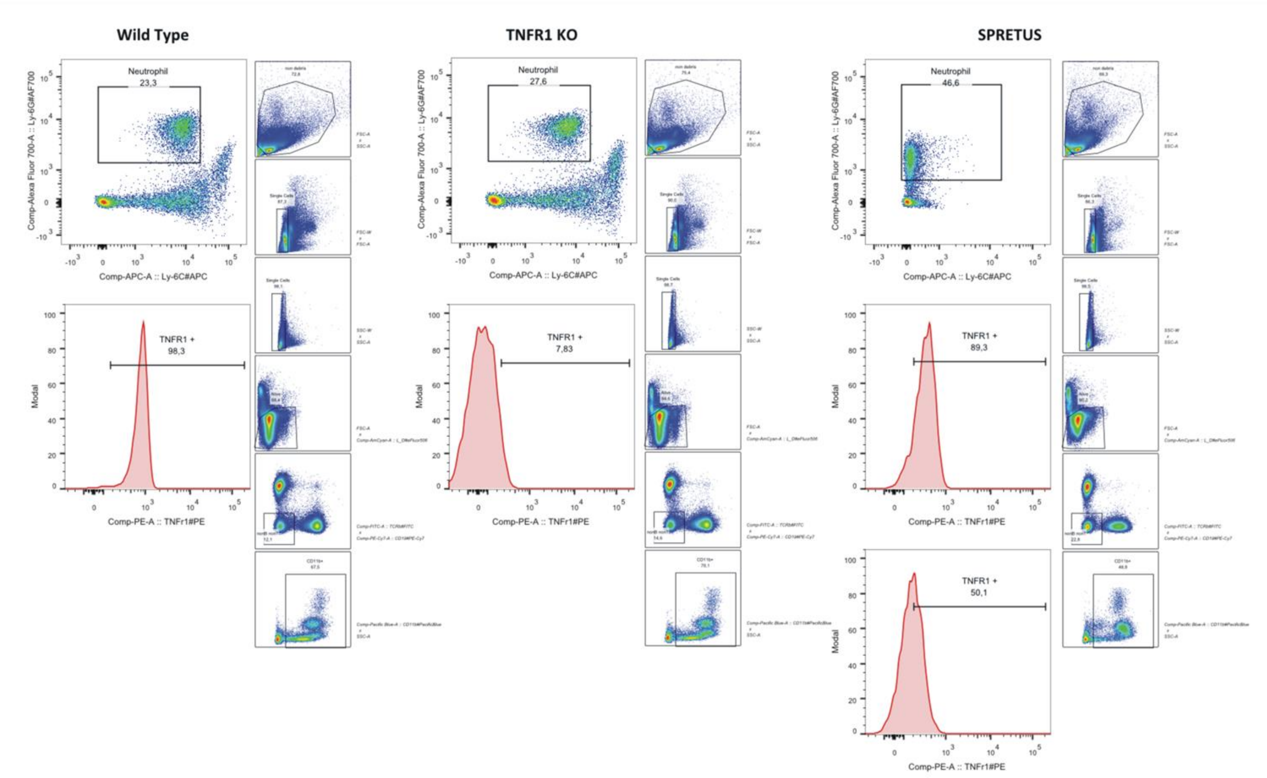
Wild Type
TNFR1 KO
SPRETUS
Neutrophil
23,3
27,6
46,6
Comp-Alexa Fluor 700-A :: Ly-6G#AF700
Comp-APC-A :: Ly-6C#APC
TNFR1 +
98,3
7,83
89,3
50,1
Modal
Comp-PE-A :: TNFr1#PE

S8

Figure	Compared groups		p-value	
1A	20µg	C57BL/6, SPRET/Ei	<0.0001	
		C57BL/6, F1	<0.0001	
		C57BL/6, TNFR1-/-	0.0004	
		C57BL/6, TNFR1+/-	0.0001	
	100µg	C57BL/6, SPRET/Ei	0.0001	
		C57BL/6, F1	0.0001	
		C57BL/6, TNFR1-/-	0.0001	
		C57BL/6, TNFR1+/-	0.0001	
	20µg	BB, BS	1937	
		BB, SS	0.2526	
2A	Liver	B, +/-	0.0059	
		B, -/-	<0.0001	
		B, F1	0.0001	
		B, S	0.040	
	Kidney	B, S	0.0002	
	Lung	B, S	0.0029	
	Spleen	B, S	0.0109	
	Serum	B, S	0.0002	
	Liver	BB, SS	0.3984	
		BB, BS	0.9537	
2B		WT, -/-	<0.0001	
		WT, S	0.0013	
		-/-, S	0.0019	
2C	Liver	B, S	0.5651	
	Liver Affimetrix	B, S	0.3845	
	Kidney	B, S	0.5191	

	**Lung**	**B, S**	**0.7229**	
	**Spleen**	**B, S**	**0.3399**	
**3A**	**Liver**	**miR-511**	**0.0458**	
		**Mrc1**	**0.0116**	
	**Spleen**	**miR-511**	**0.0473**	
		**Mrc1**	**0.0275**	
**4A**	**Left**	**3'UTR only, miR-CTR**	**0.1626**	
		**miR-CTR, miR-511**	**<0.0001**	
		**3'UTR only, miR-511**	**<0.0001**	
	**Right**	**3'UTR only, miR-CTR**	**0.0699**	
		**miR-CTR, miR-511**	**<0.0001**	
		**3'UTR only, miR-511**	**<0.0001**	
**4B**	**B**	**miR-CTR, miR-511**	**0.048**	
	**S**	**miR-CTR, miR-511**	**0.0005**	
**4C**	**B**	**Anti-miR-CTR, anti-miR-511**	**0.0021**	
	**S**	**Anti-miR-CTR, anti-miR-511**	**0.0010**	
**4D**	**Left, TNFR1**	**PBS, miR-CTR**	**0.8207**	
		**PBS, miR-511**	**0.0318**	
		**miR-CTR, miR-511**	**0.0035**	
	**Middle, Survival**	**miR-511, others**	**0.0353**	
	**Right, Body Temperature**	**PBS, miR-CTR**	**0.1333**	
		**PBS, miR-511**	**<0.0001**	

		**miR-CTR, miR-511**	**0.0007**	
**4E**	**Left, Survival**	**miR-511, PBS**	**0.0352**	
		**mR-511, miR-CTR**	**0.0322**	
	**Right, Survival**	**mR-511, miR-CTR**	**0.5056**	
**4F**	**Left, ALT**	**PBS, miR-CTR**	**0.0511**	
		**PBS, miR-511**	**0.0014**	
		**miR-CTR, miR-511**	**0.0032**	
	**Middle, Body Temperature**	**PBS, miR-CTR**	**0.0230**	
		**PBS, miR-511**	**0.0003**	
		**miR-CTR, miR-511**	**0.0384**	
	**Right, TNF**	**PBS, miR-CTR**	**0.9170**	
		**PBS, miR-511**	**0.8914**	
		**miR-CTR, miR-511**	**0.7663**	
**5A**	**Left, TNFR1**	**PBS, anti-miR-CTR**	**0.0789**	
		**PBS, anti-miR-511**	**0.0008**	
		**Anti-miR-CTR, anti-miR-511**	**0.0418**	
	**Middle, survival**	**PBS, anti-miR-511**	**0.0008**	
		**Anti-miR-CTR, anti-miR-511**	**0.0010**	
	**Right, Body Temperature**	**PBS, anti-miR-CTR**	**0.8010**	
		**PBS, anti-miR-511**	**0.0058**	
		**Anti-miR-CTR, anti-miR-511**	**0.0156**	
**5B**	**Left, Survival**	**PBS, anti-miR-511**	**0.001**	
		**Anti-miR-CTR,**	**0.001**	

		anti-miR-511		
	Right, Body Temperature	PBS, anti-miR-CTR	0.5945	
		PBS, anti-miR-511	<0.0001	
		Anti-miR-CTR, anti-miR-511	<0.0001	
5C	Upper panel, left	B, DMSO, RU	0.0023	
		S, DMSO, RU	0.0023	
	Upper panel, right	B, DMSO, RU	<0.0001	
		S, DMSO, RU	0.0003	
		DMSO, DMSO	0.0721	
		RU, RU	0.3469	
	Lower panel, left	B, DMSO, RU	<0.0001	
		S, DMSO, RU	<0.0001	
		DMSO, DMSO	0.1448	
		RU, RU	0.5113	
	Lower panel, right	Liver, DMSO, RU	0.0280	
		Spleen, DMSO, RU	0.0430	
6A	Survival	Sham, Adx	0.0003	
	Gene Expression	Tsc22d3	0.0088	
		Dusp1	0.0003	
		Mrc1	0.0034	
		miR-511	0.0357	
	TNFR1	Liver	0.0072	
		Spleen	<0.0001	
		Serum	0.0077	
6B	Survival	PBS, DEX	0.0018	
	Gene Expression	Tsc22d3	<0.0001	
		Sgk	0.0001	
		Tat	0.0325	
		Mrc1	0.0180	
		miR-511	0.0428	
6C	Gene Expression	Tsc22d3	<0.0001	
		Sgk	0.0001	

		**Tat**	**0.0085**	
		**Mrc1**	**0.0003**	
		**Tnfrsf1a**	**0.9515**	
		**miR-511**	**0.0125**	
**6D**	**TNFR1**	**Liver**	**0.0001**	
		**Spleen**	**0.0337**	
		**Serum**	**0.0199**	
**S3**	**TNFR1**	**PBS, anti-miR-CTR**	**0.4858**	
		**PBS, anti-miR-511**	**0.0396**	
		**Anti-miR-CTR, anti-miR-511**	**0.0044**	
	**Survival**	**PBS, anti-miR-511**	**0.0383**	
	**Body Temperature**	**PBS, anti-miR-CTR**	**0.0039**	
		**PBS, anti-miR-511**	**0.0017**	
		**Anti-miR-CTR, anti-miR-511**	**0.1526**	
