# Supplementary material for: Glucocorticoid-induced microRNA-511 protects against TNF by down-regulating TNFR1
Source: EMBO Mol Med. 2015 May 20;7(8):1004–17. doi: 10.15252/emmm.201405010 (PMC4551340; doi:10.15252/emmm.201405010)
Supplement: Supplementary file 2 [file emmm0007-1004-sd2.pdf]

Glucocorticoid-induced MicroRNA-511 protects against TNF by down-regulating TNFR1

Leen Puimège, Filip Van Hauwermeiren, Roosmarijn E. Vandenbroucke, Sophie Steeland, Sara Van Ryckeghem, Jolien Vandewalle, Sofie Lodens, Lien Dejager, Sofie Vandevyver, Jan Staelens, Steven Timmermans and Claude Libert

Corresponding author: Claude Libert, Ghent University/VIB

Review timeline:

Submission date:	31 December 2014
Editorial Decision:	30 January 2015
Revision received:	15 April 2015
Editorial Decision:	24 April 2015
Accepted:	28 April 2015

Transaction Report:

(Note: With the exception of the correction of typographical or spelling errors that could be a source of ambiguity, letters and reports are not edited. The original formatting of letters and referee reports may not be reflected in this compilation.)

Editor: Céline Carret

1st Editorial Decision 30 January 2015

Thank you for the submission of your manuscript to EMBO Molecular Medicine. We have now heard back from the three referees whom we asked to evaluate your manuscript.

As you will see from the comments below, the referees are rather supportive of the study but do have suggestions to improve and strengthen the data. I will not get into details as you will find the reviews sufficiently explicit, but hope that you will be willing to submit a revision taking into account all comments from the referees.

As you know, it is EMBO Molecular Medicine policy to allow only a single round of revision and that, as acceptance or rejection of the manuscript will depend on another round of review, your responses should be as complete as possible.

We would welcome the submission of a revised version for further consideration and depending on the nature of the revisions, this may be sent back to the referees for another round of review.

Please pay attention to the editorial requirements listed below and provide these with your revision in order to not delay the process upon resubmission.

I look forward to seeing a revised form of your manuscript within 3 months, however let us know if you anticipate some delay.

***** Reviewer's comments *****

Referee #1 (Comments on Novelty/Model System):

The authors have used three established mouse models of systemic inflammatory responses to demonstrate anti-inflammatory responses of miR-511: (1) LPS-induced endotoxemia; (2) concanavalin induced acute hepatitis; and (3) administration of TNF to induce SIRS. All three mouse models are relevant for TNF-induced systemic responses in humans.

Referee #1 (Remarks):

Puimège and colleagues have used an elegant experimental genetic approach in mice to identify a novel negative regulator of tumour necrosis factor (TNF) driven systemic inflammation and pathology. Building on their previous work that has characterised the genetic basis of the well known resistance of SPRET/Ei mice against LPS and TNF induced lethal schock syndrome, they have now taken a positional candidate gene approach to identify the microRNA-511 (miR-511) as a major regulator of tumour necrosis factor receptor 1 (TNFR1) protein expression. By generating new consomic mouse lines that carry either copies of the C57BL/6J or SPRET/Ei derived TNFR1 encoding Tnfrsf1a gene in reciprocal host genomes they convincingly show that the TNFR1 is equally functional in both strains. By screening then the Tnfrsf1a gene for miRNA target sequences they identify miR-511 which interestingly maps to the previously identified second SPRET/Ei TNF resistance locus on mouse chromosome 2. To demonstrate a mechanistic link between miR-511 expression and TNF resistance of SPRET/Ei mice the following evidence is provided: i) Compared to C57BL/6J mice, SPRET/Ei mice show increased expression of miR-511 and reduced expression of the TNFR1 protein; ii) miR-511 directly targets a 3'-UTR of the Tnfrsf1a gene resulting in reduced protein expression; iii) delivery of miR-511 by hydrodynamic tail injection protects against TNF induced pathology in three different mouse models of systemic inflammation (LPS-induced endotoxemia, concanavalin induced acute hepatitis, and TNF induced SIRS); iv) blockage of miR-511 expression with a specific anti-miR-511 miRNA leads to elevated TNFR1 expression in C57BL/6J and SPRET/Ei mice and sensitizes TNF-resistant SPRET/Ei and TNF-resistant (BxS)F1 mice to otherwise non-lethal dosages of TNF. The observed increased expression of miR-511 in SPRET/Ei mice is explained by showing that the miR-511 encoding gene Mrc1 is inducible by glucocorticoids that are known to be strongly upregulated in SPRET/Ei mice due to an overactive hypothalamic-pituitary axis. Consequently, adrenalectomy of mice is shown to decrease miR-511 expression leading to sensitisation to TNF induced systemic inflammation. In line with this finding, dexamethasone treatment of mice is shown to upregulate miR-511 expression leading to reduced TNFR1 protein expression and protection against TNF-induced lethal SIRS.

This work identifies miR-511 as an important new element in the regulation of TNFR1 expression. It makes miR-511 potentially to a new drug target that might be used to inhibit specifically TNRF1 functions, a major goal of current strategies to interfere with TNF-driven acute and chronic inflammatory diseases. The study provides further novelty by showing that miR-511 is induced by glucocorticoids (GCs). This extends the well-known mechanisms of GCs in anti-inflammatory signalling by showing that through induction of miR-511 the TNF pathway is a direct target of GCs.

The study is well designed, carefully carried out and provides enough depth to support the authors conclusions. The genetic dissection of the dominant SPRET/Ei TNF resistance is technically very challenging, especially in this case, which demonstrates that a miRNA underlies the molecular nature of two QTLs. PuimËge and colleagues have successfully mastered this challenge by taken advantage of a smart bioinformatic mining approach, which makes this paper clearly outstanding from others in the field. The manuscript is well written and is technically sound. There are a few open questions that should be addressed to clarify a few issues and to further strengthen the paper.

General comments:

(1) To screen for miRNA-based trans regulation of the Tnfrsf1a gene the authors have used the MiR Walk software which provides different algorithms for identification of miRNAs target sequences. However, it is not stated which Tnfrsf1a sequences have been used for this. Have all annotated Tnfrsf1a mRNA isoforms been screened for miRNA target sequences? Is the miR-511 target site

found in all 3'-UTRs of Tnfrsf1a transcripts (e.g. possibly also in alternatively used 3'-UTRs)? This should be clarified and the information included into the manuscript.

(2) The miR-511 mediated downregulation of TNFR1 protein expression levels is convincing. Are there possibly also other gene targets of miR-511 that might contribute to the observed downregulation of the TNFR1 protein? What are other predicted targets of miR-511? If these exist would such targets possibly also crosstalk to LPS/TNF signalling pathways?

(3) Do the authors have evidence that the miR-511 mediated downregulation of TNFR1 works also in the human system? If this mechanism is conserved across species it would significantly strengthen the argument that miR-511 has therapeutic potential for selective inhibition of TNFR1 functions.

(4) Figure 2C: Has the TNFR1 protein expression trait been tested for genetic interactions between the chromosome 2 and 6 loci? It would be interesting to know if the SPRET/Ei resistance alleles on chr2 show a dominant or additive effect on the chr6 TNFR1 expression levels. From the 214 N2 cross mapping data it should be possible to infer if heterozygosity or homozygosity of SPRET/Ei alleles in the Mrc1/miR-511 locus have different effects on TNFR1 protein expression levels.

Minor criticism:

- The Mrc1 gene is not introduced on page 7. A single sentence should be included stating that this gene encodes the C type 1 mannose receptor.

- Specify in Material and Methods which bioassays have been used for measurement of serum TNF and serum IL-6 levels

- Legend to Figure 1B: Correct 'the mice had the last 40 cM of chr6 in a B homozygous way' into: After N10 generations of intercrossing resulting progenies carried at least 40 cM sized haplotypes of B homozygosity (black), BS heterozygosity (green), or S homozygosity (grey).

- Legend to Figure 3: Correct in S (lower 'blue' panels)

- Throughout the manuscript use consistently the right gene symbol for the murine Tnfrsf1a gene

- Italise all gene symbols (e.g. Mrc1 and others)

Referee #2 (Remarks):

The authors have extended their earlier studies and now, using a variety of strategies including mouse breeding and backcrossing, plasmid induced over expression of M1R511 or its neutralization, together with manipulations of the glucocorticoid system make a compelling case for the concept that SPRET/Ei mice are highly resistant to the harmful effects of infused TNF (using fairly high doses) when compared to other mouse strains. The "sepsis" studies appear to involve infusion of either LPS or TNF. The authors should qualify their conclusions, since such conclusions may not apply to other models of sepsis (cecal ligation and puncture, noninfectious sepsis, etc). Accordingly, this could restrict translation applications in humans with sepsis.

Referee #3 (Comments on Novelty/Model System):

This is an extremely well performed study employing most elegant approaches. Although some data are fully convincing about the sequence of events (GCC >>> miRNA-155 >>> reduced expression

of TNFR1 >>>> increased resistance to TNF), the extremely low modulation of TNFR1 in liver cells after miR-155 injection, associated with a major impact in terms of surviving remains puzzling.

Referee #3 (Remarks):

Leen PuimËge and colleagues have performed a most elegant demonstration and an extremely well performed study to explain the high resistant of mus spretus (S) mice to TNF as compared to mus musculus (B = C57BL/6). They end to the conclusion that glucocorticoids favor the expression of miRNA-511, which limits the expression of TNFR1 expression and thus allow an increased resistance to TNF.

Main concern
Some elements of the demonstration are highly convincing. However, others are less persuading. For example, the in vivo treatment with miR-511 only leads to a 14% decrease of TNFR1 expression in the liver (fig.4D) and not 20% as stated in the text (p.8). This rather low decrease is only occurring in the liver while one may suppose that the level of TNFR1 expression remains similar in the spleen. Since the authors are not using a toxic model that would be mainly targeting the liver (i.e. TNF + galactosamine), one may guess that the spleen does play a major role in this model. Indeed, among all tissues, spleens of S mice have the lowest expression of TNFR1 protein. Also the spleen of S mice express twice the amount of miR-511 found in B mice while it is only 30% more in the liver.

Accordingly, it is difficult to explain that such a low decrease in the liver after miR-155 treatment is sufficient to explain the significant survival improvement. Since the authors measured the total TNFR1 in the liver, it is difficult to assume that this level perfectly correlates with the membrane expression of the receptor. What about TNFR1 shedding, what about intracellular accumulation? Because anti-mouse CD120a (TNF R Type I/p55) antibodies are commercially available, a direct flow cytometry analysis of TNFR1 expression on cell surface would have been more appropriate. A comparison of CD120a expression in the spleen (and eventually liver) of B and S mice would be of great interest.

Other comments
1/ Please express endotoxin contamination of recombinant TNF /mg or /µg rather than /ml.
2/ Fig.2A. It is unclear if the whole figure is expressed as % TNFR1 expression in liver of B mice (left bar). It would be more logical to express as % in each B mice tissues (i.e. kidney and spleen). It may be the case, but then B kidney and B spleen should be given as 100%.
3/ Please use similar vertical axis for figure 3A (right and left), for figure 4B and 4C, for figure 4E (right and left).
4/ Figure 6B: Please gather on the same figure right panel and lower panel to allow comparisons with the same vertical axis. Do it as well for fig.6C left and right panel
5/ Why PBS controls of Tsc22d3, Sgk1, Tat (fig.6B right panel) are far below one?
6/ Most data are expressed as percent of control. Accordingly, it is sometimes difficult to appreciate the relative amounts from tissues to tissues: is the liver expressing more TNFR1 than the spleen (cf fig.2A, fig.5C lower right panel , fig. 6A lower panel, fig.6D)?
6/ What are the levels of cortisol after TNF injection in B and S mice?

1st Revision - authors' response 15 April 2015

Referee #1 (Comments on Novelty/Model System):

The authors have used three established mouse models of systemic inflammatory responses to demonstrate anti-inflammatory responses of miR-511: (1) LPS-induced endotoxemia; (2) concanavalin induced acute hepatitis; and (3) administration of TNF to induce SIRS. All three mouse models are relevant for TNF-induced systemic responses in humans.

Referee #1 (Remarks):

Puimège and colleagues have used an elegant experimental genetic approach in mice to identify a novel negative regulator of tumour necrosis factor (TNF) driven systemic inflammation and pathology. Building on their previous work that has characterised the genetic basis of the well known resistance of SPRET/Ei mice against LPS and TNF induced lethal schock syndrome, they have now taken a positional candidate gene approach to identify the microRNA-511 (miR-511) as a major regulator of tumour necrosis factor receptor 1 (TNFR1) protein expression. By generating new consomic mouse lines that carry either copies of the C57BL/6J or SPRET/Ei derived TNFR1 encoding Tnfrsf1a gene in reciprocal host genomes they convincingly show that the TNFR1 is equally functional in both strains. By screening then the Tnfrsf1a gene for miRNA target sequences they identify miR-511 which interestingly maps to the previously identified second SPRET/Ei TNF resistance locus on mouse chromosome 2. To demonstrate a mechanistic link between miR-511 expression and TNF resistance of SPRET/Ei mice the following evidence is provided: i) Compared to C57BL/6J mice, SPRET/Ei mice show increased expression of miR-511 and reduced expression of the TNFR1 protein; ii) miR-511 directly targets a 3'-UTR of the Tnfrsf1a gene resulting in reduced protein expression; iii) delivery of miR-511 by hydrodynamic tail injection protects against TNF induced pathology in three different mouse models of systemic inflammation (LPS-induced endotoxemia, concanavalin induced acute hepatitis, and TNF induced SIRS); iv) blockage of miR-511 expression with a specific anti-miR-511 miRNA leads to elevated TNFR1 expression in C57BL/6J and SPRET/Ei mice and sensitizes TNF-resistant SPRET/Ei and TNF-resistant (BxS)F1 mice to otherwise non-lethal dosages of TNF. The observed increased expression of miR-511 in SPRET/Ei mice is explained by showing that the miR-511 encoding gene Mrc1 is inducible by glucocorticoids that are known to be strongly upregulated in SPRET/Ei mice due to an overactive hypothalamic-pituitary axis. Consequently, adrenalectomy of mice is shown to decrease miR-511 expression leading to sensitisation to TNF induced systemic inflammation. In line with this finding, dexamethasone treatment of mice is shown to upregulate miR-511 expression leading to reduced TNFR1 protein expression and protection against TNF-induced lethal SIRS.

This work identifies miR-511 as an important new element in the regulation of TNFR1 expression. It makes miR-511 potentially to a new drug target that might be used to inhibit specifically TNRF1 functions, a major goal of current strategies to interfere with TNF-driven acute and chronic inflammatory diseases. The study provides further novelty by showing that miR-511 is induced by glucocorticoids (GCs). This extends the well-known mechanisms of GCs in anti-inflammatory signalling by showing that through induction of miR-511 the TNF pathway is a direct target of GCs. The study is well designed, carefully carried out and provides enough depth to support the authors conclusions. The genetic dissection of the dominant SPRET/Ei TNF resistance is technically very challenging, especially in this case, which demonstrates that a miRNA underlies the molecular nature of two QTLs. Puimège and colleagues have successfully mastered this challenge by taken advantage of a smart bioinformatic mining approach, which makes this paper clearly outstanding from others in the field. The manuscript is well written and is technically sound. There are a few open questions that should be addressed to clarify a few issues and to further strengthen the paper.

General comments:

(1) *To screen for miRNA-based trans regulation of the Tnfrsf1a gene the authors have used the MiR Walk software which provides different algorithms for identification of miRNAs target sequences. However, it is not stated which Tnfrsf1a sequences have been used for this. Have all annotated Tnfrsf1a mRNA isoforms been screened for miRNA target sequences? Is the miR-511 target site found in all 3'-UTRs of Tnfrsf1a transcripts (e.g. possibly also in alternatively used 3'-UTRs)? This should be clarified and the information included into the manuscript.*

We have added the following clarification to the results section and the legend of Supplemental Figure 2, of the revised manuscript. **Based on the Ensembl (www.ensembl.org) annotation, only one protein coding Tnfrsf1a transcript, the consensus CDS, has a complete 3'UTR. One more transcript with a 3'UTR is annotated as nonsense mediated decay and not taken into account. So only the transcript Tnfrsf1a-001 (ENSMUST00000032491) has to be, and has been, screened for miR-511 target site(s).**

For the interest of the reviewer:

Name	Transcript ID	bp	Protein	Biotype	CDS	UniProt	RefSeq	TSL
Tnfrsf1a-001	ENSMUST00000032491	2156	454 aa	Protein coding	Consensus CDS20550	D3YXL4 P25118 Q3U479 Q6QHF1	NM_011609 NP_035739	TSL1
Tnfrsf1a-006	ENSMUST00000144524	885	295 aa	Protein coding	CDS 5' and 3' incomplete	F7A4X5	-	TSL2
Tnfrsf1a-003	ENSMUST00000130257	390	66 aa	Protein coding	CDS 3' incomplete	D3YXL4	-	TSL5
Tnfrsf1a-004	ENSMUST00000125880	621	107 aa	Nonsense mediated decay	CDS 5' incomplete	-	-	TSL3
Tnfrsf1a-007	ENSMUST00000131434	250	No protein	Processed transcript		-	-	TSL5
Tnfrsf1a-005	ENSMUST00000134803	764	No protein	Retained intron		-	-	TSL2

The Transcript Support Level (TSL) is a method to highlight the well-supported and poorly-supported transcript models for users. The method relies on the primary data that can support full-length transcript structure: mRNA and EST alignments supplied by UCSC and Ensembl.

(2) *The miR-511 mediated downregulation of TNFR1 protein expression levels is convincing. Are there possibly also other gene targets of miR-511 that might contribute to the observed downregulation of the TNFR1 protein? What are other predicted targets of miR-511? If these exist would such targets possibly also crosstalk to LPS/TNF signalling pathways?*

Yes, there are many (n=997) targets, besides *Tnfrsf1a* mRNA, predicted to be regulated by miR-511 by prediction programs. In the revised manuscript, **we mention our search for more targets in the discussion section and refer to a list in the supplemental data, as supplemental Table 1 (ST1), with the entire list of predicted targets, which are predicted by 5/10 and 4/10 programs. We then used Ingenuity Pathway Analysis (IPA) to search for potential functional links between these targets and the TNF pathway (including TNF induction pathways and TNF signaling pathways) and the LPS pathway (LPS signaling pathway). There are some interesting potential targets (not yet experimentally validated), that may thus contribute to the anti-inflammatory effects of miR-511, besides TNFR1 regulation. One example is TRAF2.**

(3) *Do the authors have evidence that the miR-511 mediated downregulation of TNFR1 works also in the human system? If this mechanism is conserved across species it would significantly strengthen the argument that miR-511 has therapeutic potential for selective inhibition of TNFR1 functions.*

This is an interesting point. We have looked into the sequence conservation of miR-511 and target sequences, but have done no functional work in human cells. This is primarily a mouse paper. To avoid over-interpretation of our data to the human system, we have done the following. **(1) we have added a remark in the discussion that the whole miR-511-TNFR1 system has not yet been validated in human cells and that we compare the mouse (C57BL/6) and human sequences of miR-511 and target sequences in a new supplemental figure 6, SF6. (2) We show that the miR-511 seed sequence of 21 nucleotides of mouse and human are 86% identical, and differ only at three nucleotides; (3) We also show a comparison of the mouse and human miR-511 binding sites in the 3' UTR of the *Tnfrsf1a* resp. *TNFRSF1A* genes and that these are 17/20 identical (most 5' target sequence shown in Fig. 3B) and 7/19 identical (most 3' target sequence of Fig. 3B). Thus the 5' most target sequence is very similar in mouse and human. (4) Finally, we show an alignment of human miR-511 and both target sequences and see that in the human case, the miR-511 hybridizes with 12/21 nucleotides to the target sequence, versus 15/21 in mouse. We write in supplemental data that indeed the experimental validation of the miR-511-TNFR1 axis has to be done before extrapolation to the human system is possible.**

(4) *Figure 2C: Has the TNFR1 protein expression trait been tested for genetic interactions between the chromosome 2 and 6 loci? It would be interesting to know if the SPRET/Ei resistance alleles on chr2 show a dominant or additive effect on the chr6 TNFR1 expression levels. From the 214 N2 cross mapping data it should be possible to infer if heterozygosity or homozygosity of SPRET/Ei alleles in the Mrc1/miR-511 locus have different effects on TNFR1 protein expression levels.*

We have studied this interesting point and found indeed significant epistatic interaction between both loci. We have added the following text to the results section. **When studying epistatic interaction between the chr.6 and chr.2 loci in terms of TNFR1 protein regulation trait, we found (1) that the N2 mice with low TNFR1 protein level in liver (<300 ng/mg protein) that were BS heterozygous for distal chr. 6 (D6Mit113), significantly more mice were BS on proximal chr.2 (D2Mit359) (n=50) than BB (n=26)(p=0.0487, chi^2) and (2) that the N2 mice with high TNFR1 protein level in liver (>300 ng/mg protein) that were BB heterozygous for distal chr. 6 (D6Mit113), significantly more mice were BB on proximal chr.2 (n=36) than BS (n=14)(p=0.0241, chi^2).**

Minor criticism:

- The Mrc1 gene is not introduced on page 7. A single sentence should be included stating that this gene encodes the C type 1 mannose receptor.

This has been corrected.

p7: MiR-511, located in intron 5 of the *Mrc1* gene, has been suggested to be strictly co-expressed with *Mrc1* encoding the C type 1 mannose receptor (Squadrito et al, 2012).

- Specify in Material and Methods which bioassays have been used for measurement of serum TNF and serum IL-6 levels

This has been corrected.

p15: … the bio-activity of TNF in the serum was measured with an MTT cell death assay using the sensitive L929 cell line. (Mosmann, T. 1983. Rapid colorimetric assay for cellular growth and survival: application to proliferation and cytotoxicity assays. J Immunol Methods 65:55-63)
p15: Serum IL-6 was measured using a specific bio-assay based on the proliferating effect of IL-6 on 7TDI hybridoma cells.

- Legend to Figure 1B: Correct 'the mice had the last 40 cM of chr6 in a B homozygous way' into: **After N10 generations of intercrossing resulting progenies carried at least 40 cM sized haplotypes of B homozygosity (black), BS heterozygosity (green), or S homozygosity (grey)**.

This has been corrected.

*- Legend to Figure 3: Correct in S (lower '**blue**' panels)*

This has been corrected.

- Throughout the manuscript use consistently the right gene symbol for the murine Tnfrsf1a gene

This has been corrected. When talking about the protein, however, we write TNFR1

- Italise all gene symbols (e.g. Mrc1 and others)

This has been corrected.

Referee #2 (Remarks):

The authors have extended their earlier studies and now, using a variety of strategies including mouse breeding and backcrossing, plasmid induced over expression of M1R511 or its neutralization, together with manipulations of the glucocorticoid system make a compelling case for the concept that SPRET/Ei mice are highly resistant to the harmful effects of infused TNF (using fairly high doses) when compared to other mouse strains. The "sepsis" studies appear to involve infusion of either LPS or TNF. The authors should qualify their conclusions, since such conclusions may not apply to other models of sepsis (cecal ligation and puncture, noninfectious sepsis, etc). Accordingly, this could restrict translation applications in humans with sepsis.

This is an interesting comment that has been taken at heart.
We have tested the therapeutic effects of miR-511 in TNF-induced lethal inflammation, LPS-induced lethal endotoxic shock and ConA-induced acute hepatitis, but not yet in CLP-induced sepsis or infection models. Although these tests are on the program, they have not been performed yet and lay beyond this paper. On the other hand, it is indeed premature to see our current data in light of therapeutic effects in human patients. **This is why we have adapted the text in abstract, introduction, results and discussion to avoid these far-reaching conclusions about therapeutic consequences of our findings.**

Referee #3 (Comments on Novelty/Model System):

This is an extremely well performed study employing most elegant approaches. Although some data are fully convincing about the sequence of events (GCC >>> miRNA-155 >>> reduced expression of TNFR1 >>>> increased resistance to TNF), the extremely low modulation of TNFR1 in liver cells after miR-155 injection, associated with a major impact in terms of surviving remains puzzling.

Referee #3 (Remarks):

Leen Puimège and colleagues have performed a most elegant demonstration and an extremely well performed study to explain the high resistant of mus spretus (S) mice to TNF as compared to mus musculus (B = C57BL/6). They end to the conclusion that glucocorticoids favor the expression of miRNA-511, which limits the expression of TNFR1 expression and thus allow an increased resistance to TNF.

Main concern
Some elements of the demonstration are highly convincing. However, others are less persuading.

For example, the in vivo treatment with miR-511 only leads to a 14% decrease of TNFR1 expression in the liver (fig.4D) and not 20% as stated in the text (p.8). This rather low decrease is only occurring in the liver while one may suppose that the level of TNFR1 expression remains similar in the spleen. Since the authors are not using a toxic model that would be mainly targeting the liver (i.e. TNF + galactosamine), one may guess that the spleen does play a major role in this model. Indeed, among all tissues, spleens of S mice have the lowest expression of TNFR1 protein. Also the spleen of S mice express twice the amount of miR-511 found in B mice while it is only 30% more in the liver. Accordingly, it is difficult to explain that such a low decrease in the liver after miR-155 treatment is sufficient to explain the significant survival improvement.

We are aware of this dilemma. We have decided to give some more explanation of our thoughts in the revised paper, section discussion. (1) We are convinced that the delivery method of the miR, namely by hydrodynamic tail vein injection, leads to effects in the liver only, as we found before using reporter genes (see Van Bogaert et al., JBC, 2011). (2) We have published recently that a modest decrease of TNFR1 expression of 50% (in TNFR1+/- mice) leads to complete resistance of mice to TNF-induced lethal inflammation (Van Hauwermeiren et al., JCI, 2013), and we have found (but not published), that even a reduction of up to 70-80% of TNFR1, leads to resistance to TNF-induced lethal shock. However, indeed, these reductions of TNFR1 were not liver-specific. (3) Hydrodynamic delivery of miR-511 to the liver proved to have protective effects in a liver-specific

model, namely the strictly TNF-TNFR1-dependent ConA lethal hepatitis model. The reason why we did not use the TNF + galactosamine model is because this is primarily a cell-death model rather than an inflammation model, and that this model is not sensitive to relatively small TNFR1 concentration differences. In this model, TNFR1 +/- mice are not resistant (Van Hauwermeiren et al., JCI, 2013), nor are SPRET/Ei mice (Staelens et al., PNAS, 2002). These data support that liver-specific effects of TNFR1 down-regulation has anti-inflammatory effects against systemic TNF models and liver- specific TNF models. **We have added comments about this dose effect of TNFR1, liver specificity and the choice of the ConA model over the TNF + galactosamine model in the discussion of the revised paper.**

Since the authors measured the total TNFR1 in the liver, it is difficult to assume that this level perfectly correlates with the membrane expression of the receptor. What about TNFR1 shedding, what about intracellular accumulation?

It is true that we measured total cell-associated TNFR1 expression in the tissues, by ELISA, because we had no other option. As will be clear from our answer on the next point, we also found convincingly (significantly) less membrane expression of TNFR1, in SPRET/Ei spleen, by FACS, compared to C57BL/6. These FACs data are not yet fully ruling out an accumulation of intracellular TNFR1 in SPRET/Ei cells. We have tried to address this by IHC, without success. However, it remains true that the SPRET/Ei cells clearly have significantly less membrane bound TNFR1 as measured by FACS. **We have added a comment in the discussion about this aspect.** Since soluble TNFR1 levels are also much lower in SPRET/Ei compared to C57BL/6 mice, we believe that the low tissue levels cannot be explained by increased shedding in SPRET/Ei mice. **Also about this, we have added a comment in the discussion about this aspect.**

Because anti-mouse CD120a (TNF R Type I/p55) antibodies are commercially available, a direct flow cytometry analysis of TNFR1 expression on cell surface would have been more appropriate. A comparison of CD120a expression in the spleen (and eventually liver) of B and S mice would be of great interest.

This remark by the reviewer has been addressed by measuring TNFR1 protein expression on spleen cells by FACS. The data are beautifully in line with all other measurements, and show that SPRET/Ei spleen cells express about 50% TFR1 levels compared to C57BL/6 spleen cells, while spleens from TNFR1-KO mice had some 5% expression of TNFR1. **These data are included in Fig. 2B of the revised paper and are discussed shortly in the results section.**

Other comments
1/ Please express endotoxin contamination of recombinant TNF /mg or /µg rather than /ml.

This has been corrected.
p14: The preparation contained less than 6 EU/mg of endotoxin as determined …
(TNF concentration 1 mg/ml)

2/ Fig.2A. It is unclear if the whole figure is expressed as % TNFR1 expression in liver of B mice (left bar). It would be more logical to express as % in each B mice tissues (i.e. kidney and spleen). It may be the case, but then B kidney and B spleen should be given as 100%.

This has been now been corrected and the TNFR1 expression levels of all C57BL/6 organs individually taken as 100%.

3/ Please use similar vertical axis for figure 3A (right and left), for figure 4B and 4C, for figure 4E (right and left).

This has been corrected.

4/ Figure 6B: Please gather on the same figure right panel and lower panel to allow comparisons with the same vertical axis. Do it as well for fig.6C left and right panel

This has been corrected.

5/ Why PBS controls of Tsc22d3, Sgk1, Tat (fig.6B right panel) are far below one?

This has been corrected.
The expression levels are relative to the expression of the housekeeping genes.

6/ Most data are expressed as percent of control. Accordingly, it is sometimes difficult to appreciate the relative amounts from tissues to tissues: is the liver expressing more TNFR1 than the spleen (cf fig.2A, fig.5C lower right panel , fig. 6A lower panel, fig.6D)?

This has been corrected.

7/ What are the levels of cortisol after TNF injection in B and S mice?

This has been tested in a new experiment and the results are added in the text in the section results.

B6 + PBS (n=6): 89.33 ± 28.46 ng/ml
B6 + TNF (n=6): 377,83 ± 153,15 ng/ml
SPRET/Ei + PBS (n=6): 192,66 ± 94,70 ng/ml
SPRET/Ei + TNF (n=6): 266,50 ± 84,83 ng/ml

2nd Editorial Decision 24 April 2015

Thank you for the submission of your revised manuscript to EMBO Molecular Medicine. We have now received the enclosed report from the referee asked to re-assess it. As you will see the reviewer is now globally supportive and I am pleased to inform you that we will be able to accept your manuscript pending editorial final amendments.

Please submit your revised manuscript within two weeks. I look forward to seeing a revised form of your manuscript as soon as possible.

***** Reviewer's comments *****

Referee #3 (Remarks):

The authors have appropriately addressed my comments
